# Crosstalk between *KIF1C* and *PRKAR1A* in left atrial myxoma

**DOI:** 10.1038/s42003-023-05094-5

**Published:** 2023-07-14

**Authors:** Mengchen Zhou, Yan Yao, Xiangyi Wang, Lingfeng Zha, Yilin Chen, Yanze Li, Mengru Wang, Chenguang Yu, Yingchao Zhou, Qianqian Li, Zhubing Cao, Jianfei Wu, Shumei Shi, Dan Jiang, Deyong Long, Jiangang Wang, Qing Wang, Xiang Cheng, Yuhua Liao, Xin Tu

**Affiliations:** 1 Department of Cardiology, Union Hospital, Tongji Medical College, Huazhong University of Science and Technology, Wuhan, 430022 China; 2 Key Laboratory of Molecular Biophysics of Ministry of Education, College of Life Science and Technology, Center for Human Genome Research, Cardio-X Institute, Huazhong University of Science and Technology, Wuhan, 430074 China; 3 National Demonstration Center for Experimental Basic Medical Education, School of Basic Medicine, Tongji Medical College, Huazhong University of Science and Technology, Wuhan, 430030 China; 4 Department of Cardiology, Beijing Anzhen Hospital, Capital Medical University, Beijing, 100029 China; 5 Department of Cardiac Surgery, Beijing Anzhen Hospital, Capital Medical University, Beijing, 100029 China

**Keywords:** Oncogenesis, Gene regulation

## Abstract

Cardiac myxoma (CM) is the most common benign cardiac tumor, and most CMs are left atrial myxomas (LAMs). Six variations of *KIF1C*, c.899 A > T, c.772 T > G, c.352 A > T, c.2895 C > T, c.3049 G > A, and c.*442_*443dup in left atrial myxoma tissues are identified by whole-exome sequencing (WES) and Sanger sequencing. RNA-seq and function experiments show the reduction of the expression of *KIF1C* and *PRKAR1A* caused by rare variations of *KIF1C*. KIF1C is observed to be located in the nucleus, bind to the promoter region of *PRKAR1A*, and regulate its transcription. Reduction of *KIF1C* decreases *PRKAR1A* expression and activates the PKA, which causes an increase in ERK1/2 phosphorylation and SRC-mediated STAT3 activation, a reduction of CDH1, TP53, CDKN1A, and BAX, and eventually promotes tumor formation both in vitro and in vivo. The results suggest that inhibition of *KIF1C* promotes the pathogenesis of LAM through positive feedback formed by the crosstalk between *KIF1C* and *PRKAR1A*.

## Abstract (web summary)

Six variations of *KIF1C* that lead to decreased expression levels are identified in patients with left atrial myxoma (LAM). Inhibition of KIF1C promote LAM pathogenesis through a positive feedback formed by the crosstalk between KIF1C and PRKAR1A.

## Introduction

Cardiac myxoma (CM) is the most common primary cardiac tumor and accounts for approximately 50% of all primary cardiac tumors. Most CM cases are sporadic, and a few are hereditary^1^. CM occurs in any heart chamber, and approximately 75–80% of CM is left atrial myxoma (LAM)^2,3^. The origin of CM might be mesenchymal cardiomyocyte progenitor cells, and no histologic difference was observed between heritable and sporadic CM tissue^4,5^. CM can recur in different chambers of the heart, and it has a higher recurrence rate in young men, patients with multifocal origins, and patients with a family history. Therefore, genetic screening should be done in CM patients^6,7^.

Protein Kinase cAMP-Dependent Type I Regulatory Subunit Alpha (*PRKAR1A*) is currently the only known pathogenic gene for CM, and its immunohistochemistry was recommended as a routine pathological test for CM by the World Health Organization’s Classification of Tumors of the Heart in 2015^8^. Inactivation of *PRKAR1A* can cause Carney complex type 1 (CNC1, OMIM:160980), an autosomal dominant syndrome characterized by multiple endocrine tumors, including CM. Approximately two-thirds of CNC cases are caused by variations in *PRKAR1A*^9^.

However, only 7% of CM patients in the clinic are associated with CNC, and pathogenic variations of *PRKAR1A* are usually not detected in sporadic CM patients, especially the most common clinic subtype, LAM^10^. These results suggest that more genetic risk factors and the molecular genetic mechanisms for CM or LAM need to be studied.

## Results

### Two rare variations of *KIF1C* were identified by WES and RNA-seq

**Fig. 1:**
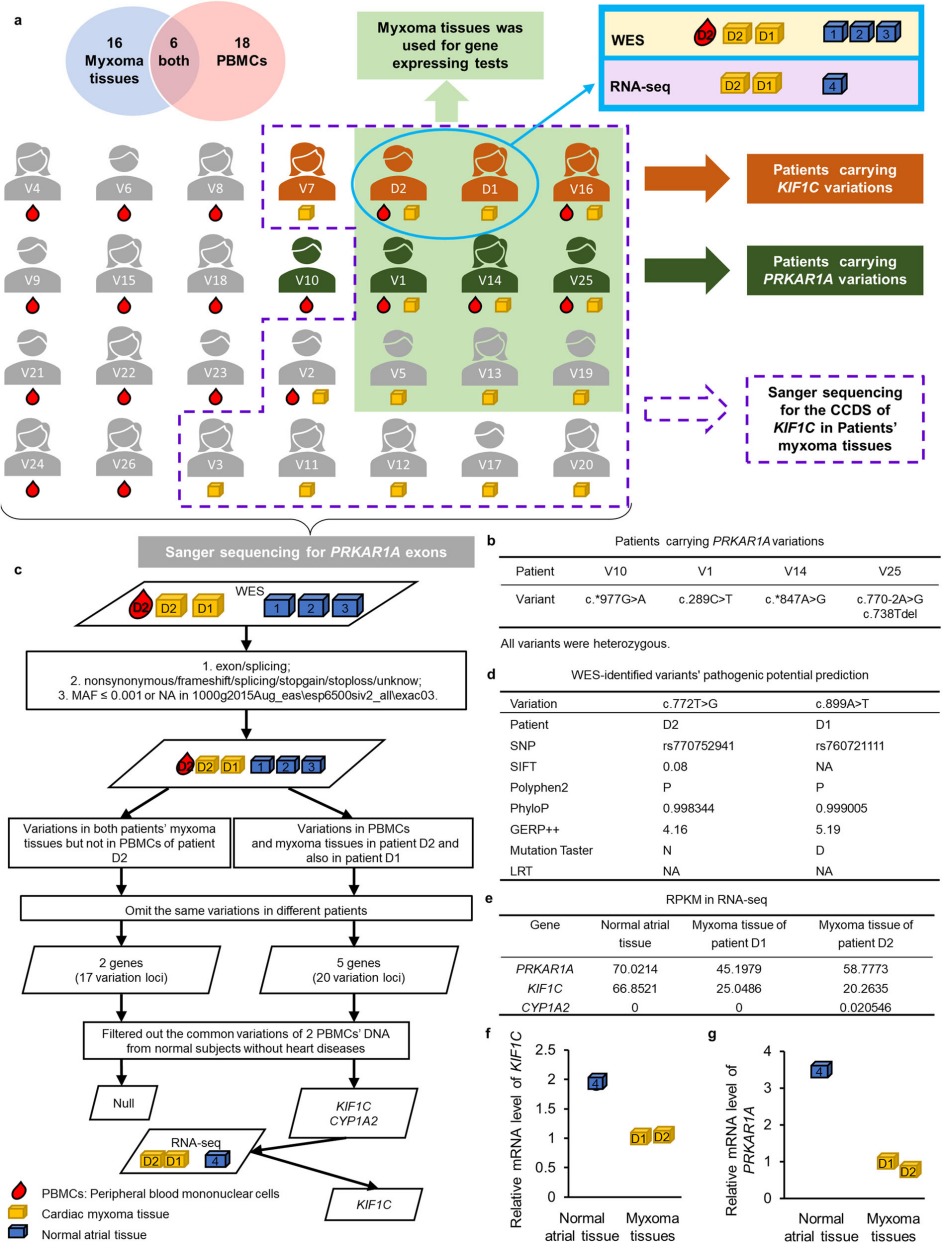
Rare variations of *KIF1C* were identified in sporadic LAM patients without pathogenic variations of *PRKAR1A*. **a** The flowchart for screening *PRKAR1A* and *KIF1C* variants. **b** Patients and their *PRKAR1A* variations. **c** Preliminary screening was performed on the variant loci list for each sample according to the following conditions: (1) the variation region was an exon or a splicing region; (2) the variation function was nonsynonymous, frameshift, splicing, stopgain, stoploss, or unknown; (3) the minor allele frequency (MAF) in the 1000g2015Aug_eas database was less than 0.001 or NA; (4) the MAF in the esp6500siv2_all database was less than 0.001 or NA; (5) the MAF in the exac03 database was less than 0.001 or NA. To identify hereditary variations that cause disease, variations in both myxoma tissues (patients D1 and D2) and PBMCs (patient D2) but not in normal atrial tissues were screened out. After eliminating systematic errors (the same variations at the same loci in different sporadic patients), five candidate genes and twenty loci were left. Two candidate genes and four loci were subjected to further study after filtering out common variations in our lab database (the WES data of two PBMCs’ DNA from normal subjects without heart diseases). To identify somatic variations that cause disease and which cannot be detected in PBMCs, variations in both myxoma tissues (patient D1 and patient D2) but neither in the PBMCs of patient D2 nor in normal atrial tissues were screened out. After eliminating systematic errors and filtering out common variations in the same way as analyzing hereditary variations that cause diseases, which was described above, no candidate genes or loci were left. **d** Pathogenic potential predictions of c.772 T > G and c.899 A > T. **e** The RPKM of RNA-seq. RPKM < 1 is not credible; RPKM < 10 represents a background expression level; 10 < RPKM < 100 represents a middle expression level. **f** The expression of *KIF1C* in the normal atrial tissue and myxoma tissues used in RNA-seq was verified by qRT-PCR. **g** The expression of *PRKAR1A* in the normal atrial tissue and myxoma tissues used in RNA-seq was verified by qRT-PCR.

Eighteen PBMCs samples and sixteen myxoma tissue samples were included in the research. A flowchart showing the experimental design, sample details, and distribution of variations is shown in Fig. 1a. Sanger sequencing for the exons of *PRKAR1A* was carried out first. Besides the previously reported c.289 C > T^7^, four variations of *PRKAR1A*: c.*847 A > G, c.*977 G > A, c.738Tdel, and c.770-2 A > G were identified (Fig. 1b, Supplementary Fig. 1, and Supplementary Table 1).

In the discovery group, based on the hypothesis that hereditary variations cause disease, two candidate genes and four loci: *CYP1A2* (NM_000761.4): c.119 A > G (rs200789139); c.1067 G > A (rs55918015); *KIF1C* (NM_006612.6): c.772 T > G (rs770752941); c.899 A > T (rs760721111) were screened out. Based on the hypothesis that somatic variations cause disease, no candidate genes or loci were screened out. The workflow of Next-Generation Sequencing (NGS) analyses is shown in Fig. 1c. The variant c.772 T > G is located in exon 9 and causes the encoded amino acid serine to be converted to alanine. The variant c.899 A > T is located in exon 11 and causes the encoded amino acid tyrosine to be converted to phenylalanine. They were predicted to be damaging by at least four of the pathogenic potential prediction algorithms: Sorting Intolerant From Tolerant (SIFT) < 0.1, Polymorphism Phenotyping v2 (PolyPhen-2) = “D” or “P”, PhyloP > 0.95, Genomic Evolutionary Rate Profiling ++ (GERP + +) > 4, MutationTaster = “A” or “D”, and Likelihood Ratio Test (LRT) = “D” (Fig. 1d). They were classified as of uncertain significance by the Clinical Interpretation of Genetic Variants (InterVar, http://wintervar.wglab.org) according to the default parameters. RNA-seq of the two myxoma tissues and a normal atrial tissue showed that the expression of *CYP1A2* was at an extremely low level without any change, while the expression of *KIF1C* was at a moderate level with an average decrease of 66% in myxoma tissues (Fig. 1e). And they were all verified by qRT-PCR (Fig. 1f, g, and Supplementary Table 2).

### More loss-of-function variations of *KIF1C* were identified in sporadic LAM patients

**Fig. 2:**
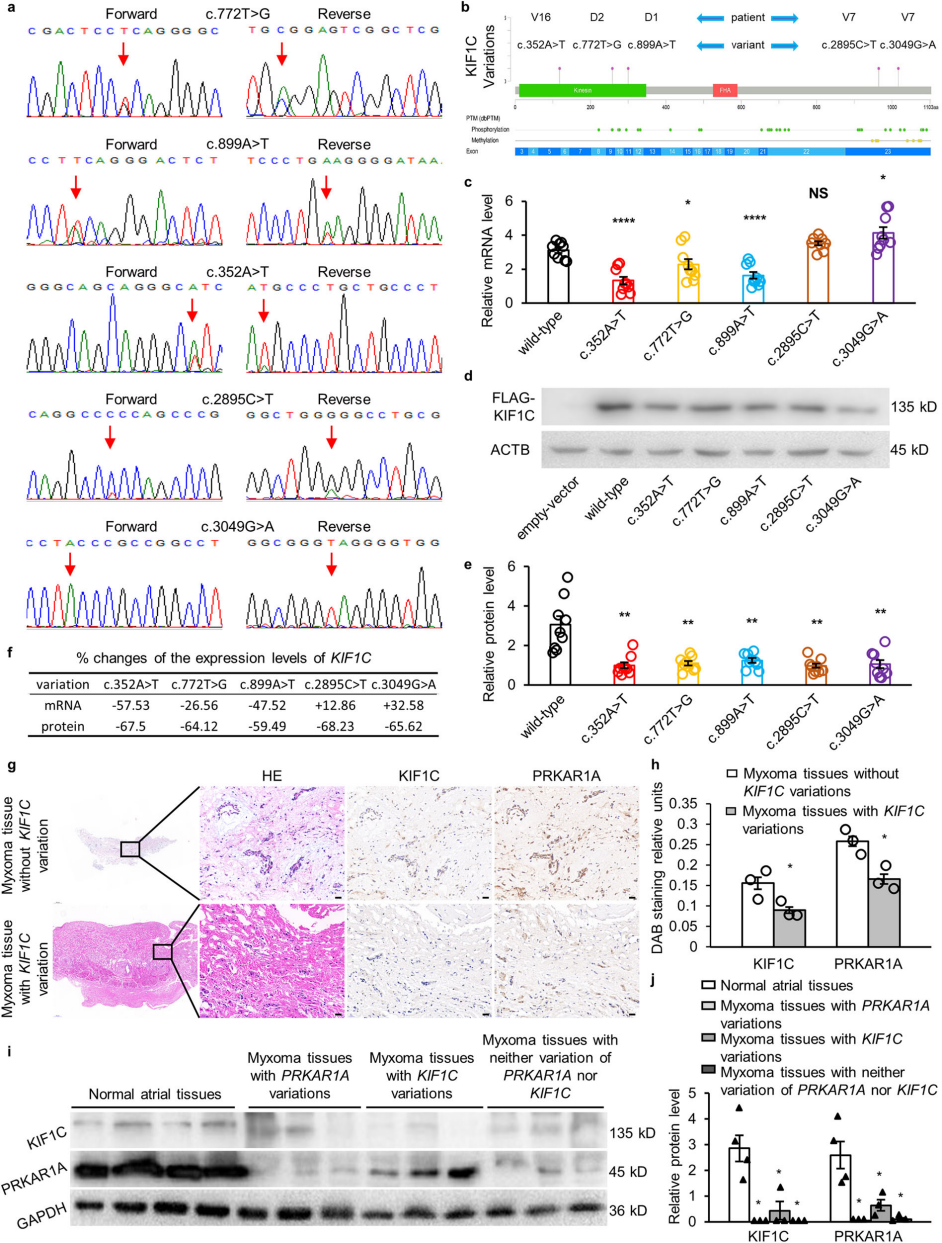
More loss-of-function variations of *KIF1C* were identified in sporadic LAM patients. **a** Sanger sequencing of c.352 A > T, c.772 T > G, c.899 A > T, c.2895 C > T, and c.3049 G > A. **b** The distribution of *KIF1C* variations in CCDS identified by Sanger sequencing. **c** The mRNA level of *KIF1C* in HCT116 cells transfected with wild-type plasmids or variation expression plasmids (c.2895 C > T group: *n* = 8, others: *n* = 9). **d** The expression level of *KIF1C* in HCT116 cells transfected with wild-type plasmids or variation expression plasmids. **e** Statistical analysis of protein quantification of *KIF1C* in HCT116 cells transfected with wild-type plasmids or variation expression plasmids (*n* = 9). **f** The percentage changes in the mRNA and protein levels of *KIF1C*. **g** HE and immunohistochemical staining for KIF1C and PRKAR1A in myxoma tissues without *KIF1C* variations and myxoma tissues with *KIF1C* variations (c.772 T > G, c.899 A > T, and c.352 A > T). Scale bar: 20 μm. **h** Quantitative analysis of immunohistochemical staining for KIF1C in myxoma tissues with and without *KIF1C* variations (*n* = 3). **i** Immunoblotting of the normal atrial tissues (*n* = 4), myxoma tissues with *PRKAR1A* variations (*n* = 3), myxoma tissues with *KIF1C* variations (*n* = 3), and myxoma tissues with neither variation of *PRKAR1A* nor *KIF1C* (*n* = 3). **j** Statistical analysis of protein quantification of KIF1C and PRKAR1A in the immunoblotting figures in **i**. Data are mean ± SEM. Statistical analysis was conducted only between the experimental group and the control group, using an unpaired *t* test for **c** and **e**, and a Mann–Whitney U test for **h** and **j**. Significance: *p* ≥ 0.05 = NS, ^*^p < 0.05, **p < 0.01, ***p < 0.0001.

The CCDS of *KIF1C* (CCDS11065) was sequenced in cDNA samples that were extracted from myxoma tissues in patients without *PRKAR1A* variations in the validation group. Other than c.772 T > G and c.899 A > T, four variations of *KIF1C* were identified by Sanger sequencing in LAM patients (Fig. 2a, b, and Supplementary Fig. 2): c.352 A > T in exon 5, which causes the encoded amino acid isoleucine to be converted to phenylalanine; c.2895 C > T in exon 23, where the encoded proline remains unchanged; c.3049 G > A in exon 23, which causes the encoded amino acid alanine to be converted to threonine; c.*442_*443dup in the 3’UTR. All variations identified in the CCDS of *KIF1C* and their minor allele frequency (MAF) values are shown in Table 1 and Supplementary Fig. 2. Compared to the cells transfected with wild-type plasmids, the mRNA levels of *KIF1C* in cells transfected with c.352 A > T, c.772 T > G, and c.899 A > T variation expression plasmids were decreased by 57.53%, 26.56%, and 47.52%, respectively. And the mRNA levels of *KIF1C* in cells transfected with c.2895 C > T and c.3049 G > A variation expression plasmids were increased by 12.86% and 32.58%, respectively (Fig. 2c). However, the expression of *KIF1C* was decreased by 67.5%, 64.12%, 59.46%, 68.23%, and 65.62%, respectively, in the cells transfected with the five variation expression plasmids (Fig. 2d, e). The percentage changes in the mRNA and protein levels of *KIF1C* are shown in Fig. 2f. The immunohistochemistry results showed consistent results that the average expression levels of *KIF1C* and *PRKAR1A* in myxoma tissues with *KIF1C* variations (c.772 T > G, c.899 A > T, and c.352 A > T) were decreased by 42.37% and 35.71%, respectively, relative to the myxoma tissues without *KIF1C* variations (Fig. 2g, h). And the immunoblotting figures showed the expression of *KIF1C* and *PRKAR1A* in all myxoma tissues was lower than that in normal atrial tissues (Fig. 2i, j).

**Table 1:** Rare variations in the CCDS (CCDS11065) of *KIF1C* in left atrial myxoma tissues.

No.	Nucleotide change (NM_006612.6)	Protein change (NP_006603.2)	SNV	Frequency						VT	InterVar
				Korea 1 K	TOPMED	ExAC	GnomAD_exome	GnomAD v2.1.1	GnomAD v2.1.1_East Asian		
D2	c.772 T > G^a^	p.Ser258Ala	rs770752941	5.0 × 10^−4^	1.9 × 10^−5^	3.4 × 10^−5^	3.2 × 10^−5^	3.194 × 10^−5^	4.351 × 10^−4^	Het	uncertain significance (PM1, PM2)
D1	c.899 A > T^a^	p.Tyr300Phe	rs760721111	-	-	4.9 × 10^−5^	3.6 × 10^−5^	3.535 × 10^−5^	4.512 × 10^−4^	Het	uncertain significance (PM1, PM2, PP3)
V16	c.352 A > T	p.Ile118Phe	-	-	-	-	-	-	-	Het	uncertain significance (PM1, PM2, PP3)
V7	c.2895 C > T	p.Pro965Pro	-	-	-	-	-	-	-	Het	likely benign (PM2, BP4, BP7)
V7	c.3049 G > A	p.Ala1017Thr	rs185185243	0.0131	7.37 × 10^−4^	1.265 × 10^−3^	1.4 × 10^−3^	1.316 × 10^−3^	0.01597	Hom	likely benign (BS1, BP4)

a, Discovered by NGS and verified by Sanger sequencing; VT, Variation Type; Het, Heterozygous; Hom, Homozygous.

### Knockdown of *KIF1C* promoted tumor growth both in vitro and in vivo

**Fig. 3:**
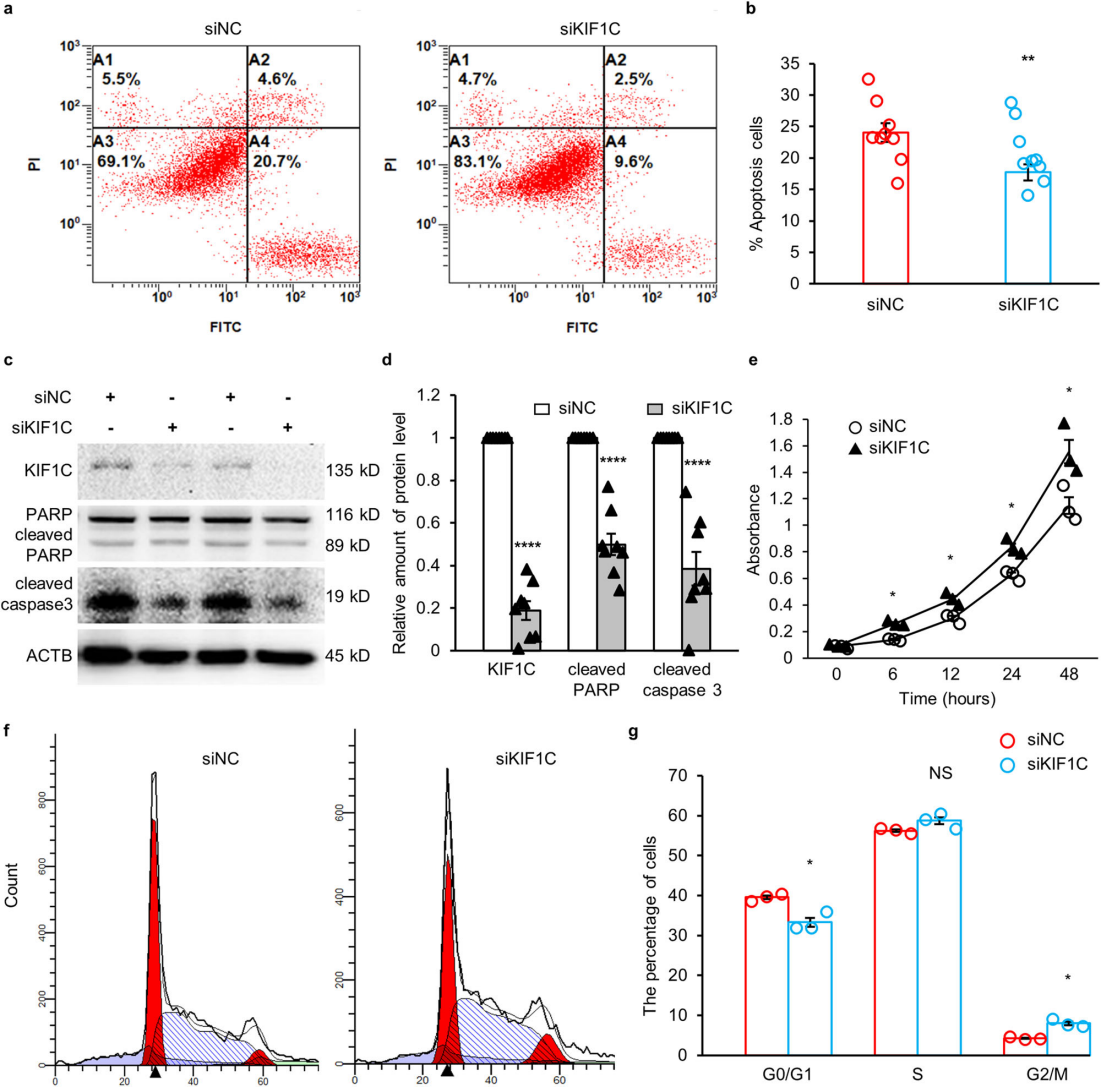
*KIF1C* knockdown promoted tumor growth at the cellular level. **a** Apoptosis was measured by FITC-Annexin-V/PI staining. **b** Statistical analysis of the apoptosis rate (*n* = 9). **c** Immunoblotting of the cleaved caspase 3 and cleaved PARP. **d** Statistical analysis of protein quantification of cleaved caspase 3 (*n* = 8) and cleaved PARP (*n* = 8). **e** Absorbance at 450 nm of cells incubated with CCK-8 for one hour (*n* = 3). **f** Cell cycle distribution was assessed by flow cytometry. **g** Statistical analysis of cell percentages in G0/G1, G2/M, and S phases (*n* = 3). Data are mean ± SEM. Statistical analysis was conducted only between the experimental group and the control group, using an unpaired *t* test for **b** and **d**, and a Mann–Whitney U test for **e** and **g**. Significance: *p < 0.05, **p < 0.01, ****p < 0.0001 .

CM and colonic carcinoma are both cell-proliferative phenotypes of CNC^11^. Considering the limitations of the few patients we accept each year, the origin cell of CM is still not clear, colon cancer tumors and CM are both polypoid, mucinous adenocarcinomas, and the human colon cancer cell HCT116 is prone to tumor formation, so HCT116 was chosen as the subject. RNAi was used to knock down *KIF1C*. After 6 h of stimulation with 150 μM H_2_O_2_, the average apoptotic rate for siKIF1C-transfected cells was reduced by 6.31% (Fig. 3a, b). Cleaved PARP was decreased by 50%, and cleaved caspase 3 was decreased by 62% when *KIF1C* was decreased by more than 80% (Fig. 3c, d). The cell viability assay showed that the viability of siKIF1C-transfected cells was increased (Fig. 3e). In siKIF1C-transfected cells, the number of cells in the G0/G1 phase decreased by 6.28%, and the number of cells in the G2/M phase increased by 3.77%, suggesting that cell proliferation was accelerated (Fig. 3f, g).

**Fig. 4:**
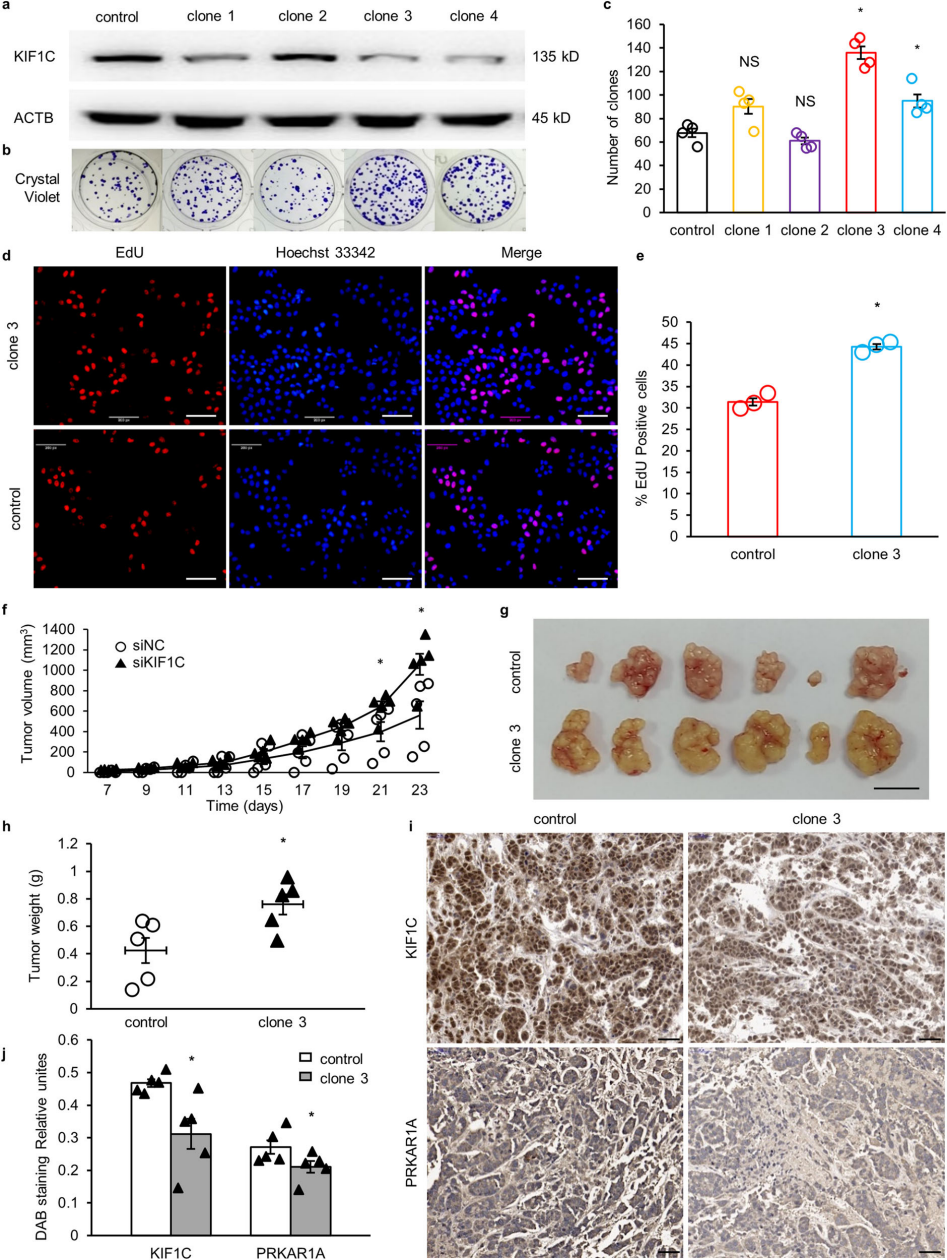
*KIF1C* knockdown promoted tumor growth in vivo. **a** Immunoblotting of the KIF1C in different monoclonal cells. **b** Crystal violet staining of colony formation from different monoclonal cells. **c** Statistical analysis of the number of clones formed by different monoclonal cells (*n* = 4). **d** Confocal microscopy of immunofluorescent staining for EdU. Nuclei were stained with Hoechst 33342. Scale bar: 200 px. **e** Statistical analysis of the EdU positive cell percentages (*n* = 3). **f** The growth curve of xenograft tumors from mice inoculated with control cells or *KIF1C* stable knockdown clone 3 cells (*n* = 5). **g** Xenograft tumors were stripped and imaged. Scale bar: 15 mm. **h** The weight of xenograft tumors from mice inoculated with control cells or *KIF1C* stable knockdown clone 3 cells (*n* = 5). **i** Immunohistochemical staining for KIF1C and PRKAR1A in xenograft tumor tissues. Scale bar: 50 μm. **j** Statistical analysis of immunohistochemical staining for KIF1C and PRKAR1A in xenograft tumor tissues (*n* = 5). One extreme data point was omitted in each group of xenograft tumors. Data are mean ± SEM. Statistical analysis was conducted only between the experimental group and the control group, using the Mann-Whitney U test. Significance: *p* ≥ 0.05 = NS, *p < 0.05, **p < 0.01.

The *KIF1C* stable knockdown cell line clone 3 was selected as the study subject because it had the lowest expression level of *KIF1C* and formed the most monoclones (Fig. 4a, b, c). The increase in the number of EdU-positive cells suggested that clone 3 cells proliferated faster than control cells (Fig. 4d, e). An equal number of control cells and clone 3 cells were individually inoculated into the right armpits of BALB/c-nude mice. A week later, tumors began to appear. The size of each tumor was measured every other day until the mice were sacrificed. The average volume of the tumor from the mice inoculated with clone 3 cells was 1.9-fold that of the control group (Fig. 4f, g). The average weight of the stripped xenograft tumors from mice inoculated with clone 3 cells was approximately 1.8 times that of the control group (Fig. 4h). The immunohistochemical staining results showed that the average expression level of *KIF1C* and *PRKAR1A* in the stripped xenograft tumors from mice inoculated with clone 3 cells was 51% and 60% of that of the control group, respectively (Fig. 4i, j).

### *KIF1C* regulated the expression of *PRKAR1A* at the transcriptional level

**Fig. 5:**
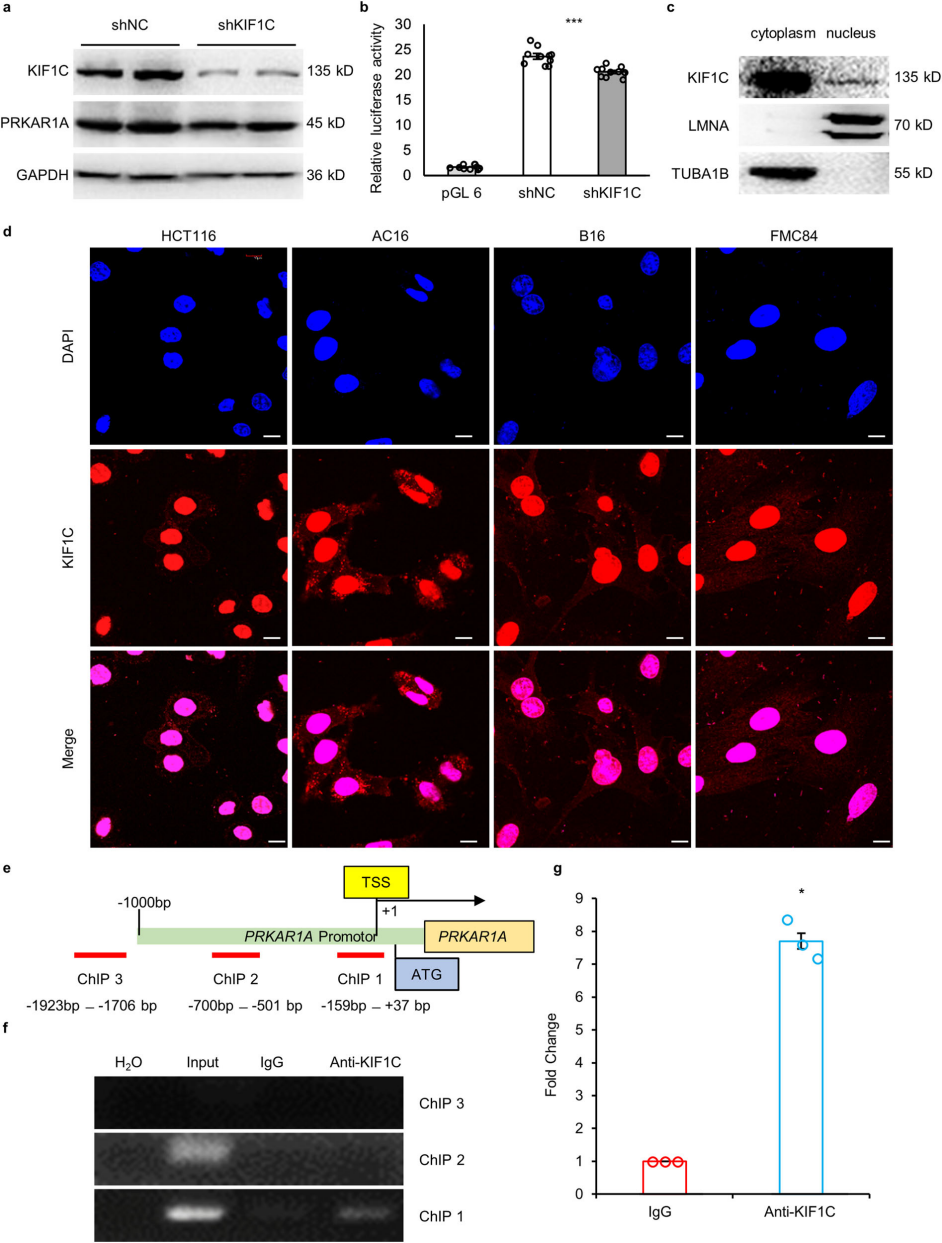
*KIF1C* was located in the nucleus, bound to the promotor region of *PRKAR1A*, and regulated *PRKAR1A* transcription. **a** Immunoblotting of PRKAR1A in *KIF1C* knockdown cells and control cells. **b** The Luciferase assay revealed *PRKAR1A* promotor activity in *KIF1C* knockdown cells and control cells (*n* = 9). **c** Immunoblotting of the endogenous KIF1C in the nucleus and cytoplasm of HCT116 cells. **d** Confocal microscopy of immunofluorescent staining for KIF1C in HCT116, AC16, B16, and FMC84 cells. Nuclei were stained with DAPI. Scale bar: 10 μm. **e** Schematic diagram for ChIP-PCR. **f** Agarose gel electrophoresis pictures for ChIP-PCR products. **g** Statistical analysis of qRT-PCR results for ChIP products (*n* = 3). Data are mean ± SEM. Statistical analysis was conducted only between the experimental group and the control group, using an unpaired *t* test for **b**, and a Mann-Whitney U test for **g**. Significance: *p < 0.05, ***.*p* < 0.001.

Immunoblotting showed the expression level of *PRKAR1A* was decreased when *KIF1C* was knocked down by the shKIF1C expression plasmid (Fig. 5a). The average luciferase activity of the *KIF1C* knockdown cells decreased by 15%, showing that *KIF1C* decreased the transcriptional activity of the *PRKAR1A* promotor (Fig. 5b). KIF1C (O43896) had a nuclear localization signal (NLS) near amino acid (aa) 300: DMQSKKRKSDFIPY, according to the prediction of PSORT (https://www.genscript.com/psort.html), cNLS Mapper (http://nls-mapper.iab.keio.ac.jp/cgi-bin/NLS_Mapper_form.cgi), NLStradamus (http://www.moseslab.csb.utoronto.ca/NLStradamus), and NucPred (https://nucpred.bioinfo.se/cgi-bin/single.cgi). Besides, PSORT, cNLS Mapper, and NucPred all predicted another NLS near aa 700: PSSGKRRAPRR. The pictures of immunoblotting showed that the expression of endogenous *KIF1C* was in both the nucleus and cytoplasm (Fig. 5c). The pictures of immunofluorescence on HCT116 human colon cancer cells, AC16 human cardiomyocytes, B16 mouse melanoma cells, and FMC84 mouse cardiomyocytes showed that the endogenous KIF1C is located in both the nucleus and cytoplasm too (Fig. 5d). There were bands for both input and anti-KIF1C samples when amplified using ChIP 1 primers (Fig. 5e, f), and the average DNA content of the *PRKAR1A* promotor in the anti-KIF1C immunoprecipitated samples was 7.7-fold that of the IgG control samples (Fig. 5g), showing that KIF1C bound to the *PRKAR1A* promotor region.

### Inhibition of *KIF1C* activated downstream pathways of PKA

**Fig. 6:**
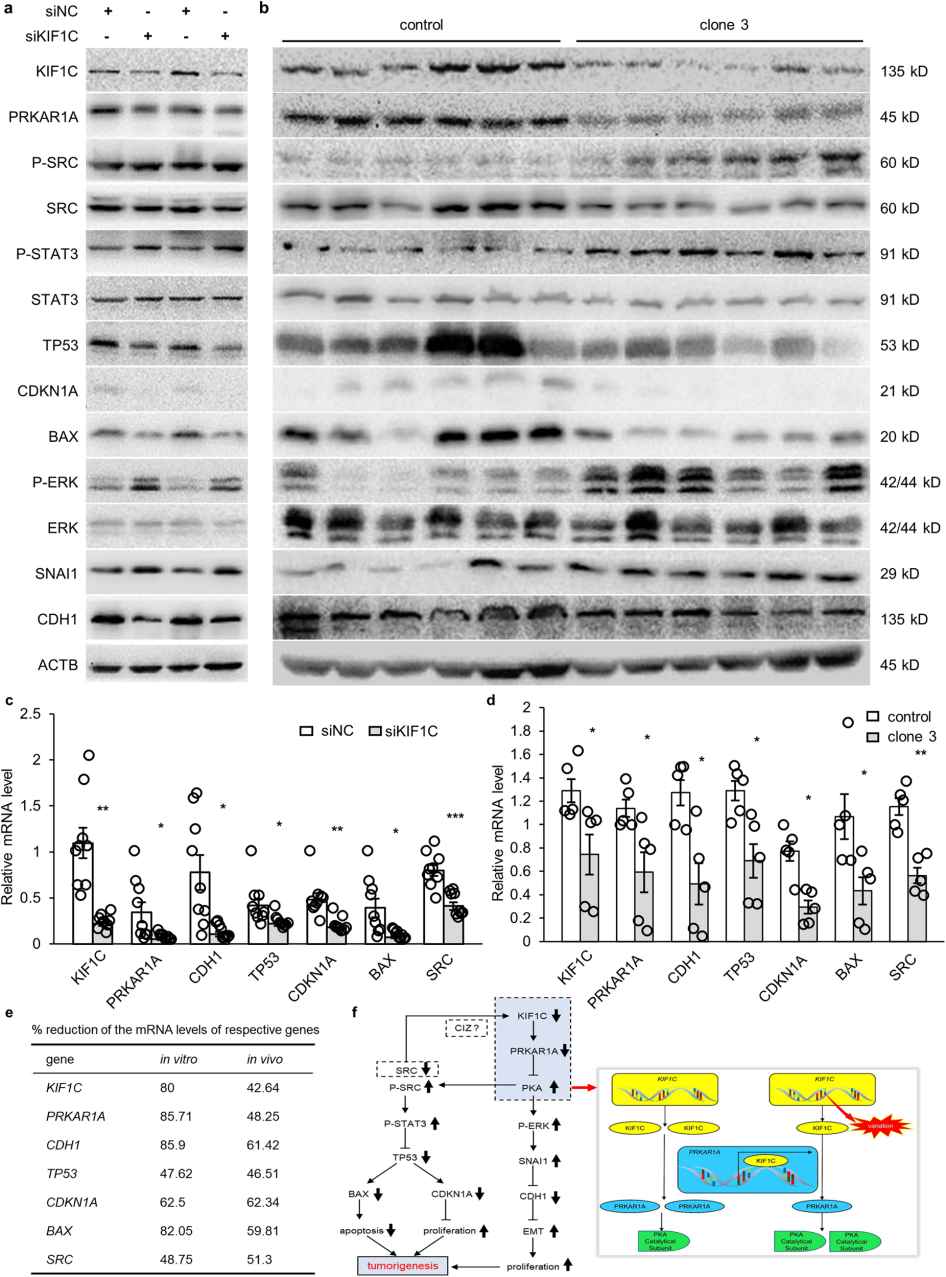
Inhibition of *KIF1C* activated downstream pathways of PKA. **a** Immunoblotting of the KIF1C, PRKAR1A, p-SRC, SRC, p-STAT3, STAT3, TP53, CDKN1A, BAX, p-ERK, ERK, SNAI1, and CDH1 in siKIF1C-transfected cells or siNC-transfected cells. **b** Immunoblotting of the KIF1C, PRKAR1A, p-SRC, SRC, p-STAT3, STAT3, TP53, CDKN1A, BAX, p-ERK, ERK, SNAI1, and CDH1 in xenograft tumors from mice inoculated with control cells or *KIF1C* stable knockdown clone 3 cells. **c** The mRNA levels of *KIF1C*, *PRKAR1A*, *CDH1*, *TP53*, *CDKN1A*, *BAX*, and *SRC* were analyzed by qRT-PCR in siKIF1C-transfected cells or siNC-transfected cells (*n* = 9). **d** The mRNA levels of *KIF1C*, *PRKAR1A*, *CDH1*, *TP53*, *CDKN1A*, *BAX*, and *SRC* were analyzed by qRT-PCR in xenograft tumors from mice inoculated with control cells or *KIF1C* stable knockdown clone 3 cells (*n* = 5). **e** The percentage reduction of the mRNA levels of *KIF1C, PRKAR1A, CDH1, TP53, CDKN1A, BAX*, and *SRC*. **f** Schematic diagram for the tumorigenesis mechanisms of the inhibition of *KIF1C* mediated by *PRKAR1A*. EMT: epithelial-mesenchymal transition. Data are mean ± SEM. One extreme data point was omitted in each group of xenograft tumors. Statistical analysis was conducted only between the experimental group and the control group, using an unpaired *t* test for **c**, and a Mann-Whitney U test for **d**. Significance: *p < 0.05, **p < 0.01, ***p < 0.001.

At the cellular level, after knockdown of *KIF1C* via siRNA, the expression level of *PRKAR1A* decreased, thus promoting excessive activation of PKA, followed by an increase in the phosphorylation of ERK, the expression of SNAI1, the phosphorylation of SRC, and the phosphorylation of STAT3, and a decrease in the expression of *CDH1*, the expression of *TP53*, the expression of *BAX*, and *CDKN1A* (Fig. 6a). Consistent immunoblotting results were observed in proteins extracted from stripped xenograft tumors (Fig. 6b).

In siKIF1C-transfected HCT116 cells, the mRNA levels of *KIF1C, PRKAR1A, CDH1, TP53, CDKN1A, BAX*, and *SRC* were decreased by 80%, 85%, 86%, 47%, 62%, 81%, and 48%, respectively (Fig. 6c). Consistent results for stripped xenograft tumors were observed: the mRNA levels of *KIF1C, PRKAR1A, CDH1, TP53, CDKN1A, BAX*, and *SRC* were decreased by 42%, 48%, 61%, 47%, 62%, 59%, and 51%, respectively (Fig. 6d). The percentage reduction of the mRNA levels of the respective genes is shown in Fig. 6e.

## Discussion

In this study, six variations of *KIF1C* were identified in patients with LAM: c.899 A > T, c.772 T > G, c.352 A > T, c.2895 C > T, c.3049 G > A, and c.*442_*443dup. At present, variations of *KIF1C* included in OMIM (https://omim.org/) are all associated with the autosomal recessive genetic disease spastic ataxia-2 (SPAX2) (OMIM: 611302). Younger CM patients were reported to have more neurologic symptoms too, such as epilepsy and pseudo-multiple sclerosis^12^. These symptoms may be related to the intracellular transport function of KIF1C^13^. The *KIF1C* variations were observed in thirty-two cancer studies in The Cancer Genome Atlas (TCGA) (Supplementary Fig. 3a), and the variation rate of the *KIF1C* is approximately 2.1%, according to statistics from the cBioPortal website (www.cBioPortal.org) (Supplementary Fig. 3b). Different types of variations all lead to decreased mRNA levels of *KIF1C* relative to normal samples, and the mRNA level of *KIF1C* in different cancer samples is also lower relative to normal samples (Supplementary Fig. 3c, d). The mRNA level of *KIF1C* is significantly reduced in human prostate cancer cells, bladder cancer cells, liver cancer cells, and melanoma cells according to the single-cell sequencing database scRNASeqDB (https://bioinfo.uth.edu/scrnaseqdb/) (GSE38495) (Supplementary Fig. 3e). Missense variations can affect mRNA splicing and/or stability and ultimately affect gene expression^14^. Consistent with these findings, in this study, all five *KIF1C* variants in CCDS had less expression compared to the wild-type in vitro. The expression of *KIF1C* was significantly decreased in the myxoma tissues of LAM patients with *KIF1C* variations (c.772 T > G, c.899 A > T, and c.352 A > T), as well as *PRKAR1A*. Besides, the expressions of *KIF1C* and *PRKAR1A* were both decreased in myxoma tissues of CM patients with *PRKAR1A* variations (c.289 C > T, c.*847 A > G, c.738Tdel, and c.770-2 A > G), which suggested that *KIF1C* and *PRKAR1A* may have upstream and downstream interactions. In addition, the expression of these two genes was decreased in myxoma tissues from patients with variations of neither *KIF1C* nor *PRKAR1A*. This result suggests that there may be other pathogenic genes for CM and that the crosstalk between *KIF1C* and *PRKAR1A* is the core pathogenic event. Interestingly, the pathogenic variations of *PRKAR1A* were observed in both atrial myxoma and ventricular myxoma, but the pathogenic variations of *KIF1C* were only observed in LAM, suggesting that variations in *KIF1C* may be pathogenic and that *KIF1C* is a new pathogenic candidate gene for CM, especially for LAM.

*KIF1C* is a member of the kinesin superfamily proteins (KIFs), which are important molecular motors involved in cell mitosis, intracellular organelles, vesicles, and protein transport^15^. Most KIFs have been found to be highly expressed in tumors and to be associated with poor prognosis, and KIFs inhibitors such as KSP/EG5 are even used for cancer treatments^16^. However, *KIF1B* and *KIF1A*, which are the most similar KIFs to *KIF1C* according to phylogenetic analysis, play different roles in tumors. *KIF1Bb* is a candidate tumor suppressor gene located at 1p36^17^. *KIF1A* exerts a protective effect and is significantly associated with 10-year recurrence-free survival in breast cancer patients^18^. *KIF1C*, *KIF1A*, and *KIF1B* are members of the UNC104 family of kinesins; they all have an FHA domain, which doesn’t exist in other KIFs besides KIF14^19^. There have been few studies on the function that the FHA domain brings to KIF1C. Many nuclear proteins containing FHA domains, like AGGF1, CHK2, and MDC1, transcriptionally regulate the expression of genes associated with cell cycle checkpoints, DNA repair, and apoptosis to achieve their tumor suppression function^20–22^. *PRKAR1A*, the only identified pathogenic gene for CM, was treated as a potent suppressor of oncogenesis in multiple tiers of immunocompetence^23^. *KIF1C* polymorphism was reported to play a role in transcriptional regulation^24^. Therefore, will *KIF1C* exert its potential tumor-suppressing function via transcriptionally regulating *PRKAR1A*?

KIF1C is widely expressed in various tissues, i.e., the brain, skeletal muscle, heart, and intestine, and in various cell types, i.e., 293 cells, NIH3T3 cells, and native C2C12 cells. It is mainly located in the cytoplasm, especially the Golgi apparatus close to the nucleus^25^. In this study, immunofluorescence and immunoblotting revealed the nuclear location of KIF1C. The Luciferase assay and ChIP assay showed that KIF1C bound to the *PRKAR1A* promoter region and regulated the expression of *PRKAR1A* at the transcriptional level.

Loss-of-function variations in *PRKAR1A* cause PRKAR1A haploinsufficiency, which affects the PKA pathway and promotes the occurrence of CM^26^. Lung adenocarcinoma is similar to CM, both of which are mucinous tumors. The expression of *PRKAR1A* is downregulated in lung adenocarcinomas. PKA activates the ERK pathway to upregulate the expression of SNAI1, SNAI1 binds to the E-box consensus sequence in the *CDH1* promoter, inhibits the expression of *CDH1*, promotes epithelial-mesenchymal transition (EMT), and eventually increases the proliferation and migration of lung adenocarcinoma cells^27^. Similarly, when *KIF1C* was knocked down in HCT116 cells, decreased mRNA and protein levels of *PRKAR1A*, increased downstream phosphorylation of ERK, and decreased mRNA and protein levels of *CDH1* were observed in this study. In addition, another pathway was observed: activated PKA increased the phosphorylation of SRC, leading to an increase in tyrosine phosphorylation of STAT3, and then, activated STAT3 binds to the promoter of *TP53*, inhibits *TP53* transcription, and decreases the expression of *TP53*. A decrease in the expression of *TP53* leads to a decrease in the expression of proapoptotic *BAX* and proliferation-inhibiting *CDKN1A*, thereby decreasing apoptosis and increasing cell proliferation at the same time. Besides, the mRNA and protein levels of *SRC* were decreased in cells when *KIF1C* was knocked down. And the mRNA and protein levels of *KIF1C*, in turn, are decreased when SRC is decreased^28^, which creates a positive feedback loop between *KIF1C* and *PRKAR1A*. Tumor formation experiments in nude mice showed that knockdown of *KIF1C* promoted tumor growth. By examining mRNA and protein levels in xenograft tumors, the tumorigenic signaling pathways described above in cells were also observed (the schematic diagram is shown in Fig. 7e).

In conclusion, variations of *KIF1C* decreased its expression, and crosstalk between *KIF1C* and *PRKAR1A* led to a positive feedback loop that enhanced the increase in cell proliferation and reduction in apoptosis, and ultimately promoted the occurrence of LAM. Multiple transcription factor JUNB binding sites are predicted in the *PRKAR1A* promoter region by JASPAR (http://jaspar.genereg.net/). One of the binding sites was located in the DNA fragment in our ChIP samples. GeneMANIA (http://genemania.org/) showed the physical interaction between KIF1C and JUNB. JUNB is a transcription factor homologous to JUN, and JUN is specifically expressed in cancer tissues. Whether KIF1C interacts with JUNB as a cofactor of the transcription factor needs to be further studied. *KIF1C* is located at 17p13.2 and *PRKAR1A* is located at 17q24.2; crosstalk between *KIF1C* and *PRKAR1A* needs to be further explored at the genetic and physiological level. Whether KIF1C directly participates in tumor-related downstream signal transduction through the strictly conserved phosphorylated threonine residues of the FHA domain is also worthy of study.

## Methods

### Study population and next-generation sequencing (NGS) analysis

Samples from twenty-eight CM patients were collected from November 2014 to May 2016 for this study. These patients were diagnosed by preoperative echocardiography and postoperative pathological examination (without other CNC symptoms). This study was conducted in accordance with the ethical principles that have their origin in the Declaration of Helsinki. Written informed consent was obtained from all subjects according to the research protocol approved by the Ethical Review Board of Beijing Anzhen Hospital (2022111X).

Two LAM patients were served as the discovery group (Supplementary Table 3), and another twenty-six patients with CM were served as the validation group (Supplementary Table 4). WES and RNA-seq were performed and annotated by Guangzhou RiboBio Co., Ltd. After pathogenic variations of *PRKAR1A* were ruled out, myxoma tissues of patients D1 and D2 and PBMCs of patient D2 were subjected to WES, and three normal atrial tissues were served as controls to get the list of variant loci for each sample. Further analysis of hereditary variations causing disease or somatic variations causing disease was carried out to identify candidate variants. The candidate variants were verified by Sanger sequencing. The RNA was extracted from the two myxoma tissues (patients D1 and D2) for subsequent RNA-seq, and another normal atrial tissue served as a control to get the differentially expressed genes for further analysis. The expression or abundance of genes was represented by RPKM (Reads Per Kilobase per Million Mapped Reads). The expression of candidate genes was verified by qRT-PCR.

### Plasmids

The sequence of *KIF1C*-targeted siRNA (siKIF1C) was 5′ CCG UCU UUA CCA UCG UCU UTT 3′, which was used to construct the plasmids pGPU6/GFP/Neo-shKIF1C (referred to as pshKIF1C in the text) and pLVX-shKIF1C-puro (referred to as pLVX-shKIF1C in the text). The negative control siRNA (siNC) sequence was 5′ UUC UCC GAA CGU GUC ACG UTT 3′, which was used to construct the plasmids pGPU6/GFP/Neo-shNC (referred to as pshNC in the text) and pLVX-shNC-puro (referred to as pLVX-shNC in the text). The promoter of *PRKAR1A* was amplified from human peripheral blood genomic DNA and used to construct the plasmid pGL6-*PRKAR1A* promotor. The CCDS of *KIF1C* was amplified from HCT116 cell cDNA and used to construct the *KIF1C* wild-type expression plasmid and five variant expression plasmids based on pCMV-3×Flag. All recombinant plasmids were confirmed by Sanger sequencing. The primers are described in Supplementary Table 5.

### Real-time quantitative PCR (qRT-PCR)

Total RNA was extracted and reversed into cDNA by M-MLV (H-) reverse transcriptase. The AceQ^®^ qPCR SYBR Green Master Mix was used to prepare the qRT-PCR reactions, and the StepOne Plus real-time PCR system was used to perform PCR. The results were analyzed with StepOne software. The relative expression of each gene was calculated by the 2^-ΔΔCt^ method. The primers are described in Supplementary Table 5.

### Cell culture, transfection, and treatment

The HCT116 cell line preserved in our laboratory was previously used for tumor study^20^. It was identified by STR and tested for the absence of mycoplasma contamination. Cells were maintained in DMEM medium supplemented with 10% fetal bovine serum in 5% CO_2_ and 21% O_2_ at 37 °C. Lipofectamine^TM^ 2000 and R4000 were used to transfect cells with plasmid DNA and siRNA, respectively. The cells were stimulated with 150 µM H_2_O_2_ for 6 h to induce apoptosis.

### Cell viability assay

Cells were incubated with CCK-8 for 1 h, and the absorbance at 450 nm was detected. The average absorbance of three replicate wells was obtained for 0 h, 6 h, 12 h, and 48 h after cells were transfected, and three independent replicative experiments were performed.

### Construction of *KIF1C* stable knockdown cell lines

A lentiviral system was used to establish a *KIF1C* stable knockdown cell line. Briefly, the pLVX-shNC or pLVX-shKIF1C, the psPAX2, and the pMD2.G plasmids were co-transfected into 293 T cells. Then the filtered supernatant was added to HCT116 cells in combination with 5 µg/mL polybrene. After being replaced with fresh complete medium, the cells were selected with 10 µg/mL puromycin and seeded in the wells of a 96-well plate at the concentration of only one cell per well by the infinite dilution method. Fourteen days later, the green single clones were selected under a fluorescence microscope. 200 control cells or *KIF1C* stable knockdown cell line cells were seeded in one well of a 24-well plate (4 replicate wells for each cell line). After culturing for two weeks, the cells were stained with crystal violet, single clones were observed, and pictures were taken.

### Cell proliferation assay

The same number of *KIF1C* stable knockdown clone 3 cells and control cells were seeded in a single well of a 24-well plate covered with a round cover glass previously. Forty-eight hours later, according to the instructions of the Cell-Light^TM^ EdU Apollo567 In Vitro Kit, the slides were sealed, observed, and imaged with an 80i fluorescence microscope.

### Flow cytometric analysis

Transfected and treated cells were proceeded according to the instructions of the FITC Annexin V Apoptosis Detection Kit and examined on an FC500 flow cytometer, and the results were analyzed with CXP software.

The same number of HCT116 cells were seeded in a six-well plate and transfected. After 48 h, the cells were stained with propidium iodide (PI) and examined on the same flow cytometer. The results of three independent experiments were analyzed using ModFit software.

### Immunoblotting

Cells and tissues grounded with a homogenizer were lysed with precooled Western and IP lysis buffer. A nuclear and cytoplasmic extraction kit was used to extract nucleoprotein and cytoplasmic proteins from cells. The BCA method was used to quantify the protein. The proteins were mixed with loading buffer, and equal amounts of proteins were loaded onto Tris-glycine SDS-PAGE gels. After electrophoresis, the PVDF membranes were blocked and incubated with primary antibodies and secondary antibodies, and the blots were visualized and analyzed with Quantity One software. Membranes that needed to be re-probed were rinsed in stripping buffer. The used antibodies are described in Supplementary Table 6.

### Immunofluorescence

Cells were seeded in a 24-well plate covered with a round cover glass per well. When the cell density reached 80%, the cells were fixed, permeabilized, blocked, incubated with a primary antibody against KIF1C, incubated with a TRITC-labeled secondary antibody, and then stained with 1 µg/mL DAPI. The sealed slides were observed using a FV1000 laser confocal microscope, and pictures were taken.

### Tumor growth assays in vivo

The mouse studies were approved by the Ethical Review Board of Beijing Anzhen Hospital. Twelve 4- to 5-week-old BALB/c-nude female mice were housed under specific pathogen-free (SPF) conditions and randomly divided into two groups. Control cells or clone 3 cells were resuspended in 0.2 mL PBS at a concentration of 5 × 10^6^ cells/mL (viable cell count >90%) and subcutaneously inoculated into the right armpit of each mouse in each group. A week later, tumors began to appear. The size of each tumor was measured every other day. Until the 23rd day, the mice were anesthetized by intraperitoneal injection of an excess of sodium pentobarbital (240 mg/kg) to sacrifice. The tumors were stripped, and the weight of each tumor was measured. The tumor volume was calculated according to the formula 0.5 × long diameter × short diameter × short diameter.

### Luciferase assay

HCT116 cells were seeded in a 24-well plate and co-transfected with pGL6/pGL6-*PRKAR1A* promoter, pshNC/pshKIF1C, and pRL-TK. 48 h later, the cells were subjected to a luciferase assay using a Dual-Glo^®^ Luciferase Assay System. The fluorescence intensities of firefly and Renilla were measured with a Glomax 20/20 luminometer.

### ChIP assay

Cells were processed according to the instructions of the SimpleChIP^TM^ Enzymatic Chromatin IP Kit. Primers targeting different regions upstream of the *PRKAR1A* were designed for standard PCR and qRT-PCR. The amplified products were electrophoresed on a 1% agarose gel with GoldView^TM^ nucleic acid dye.

### Statistics and reproducibility

Pairwise comparisons between the experimental group and the control group were performed using unpaired *t* tests in Excel and the Mann–Whitney U test in SPSS (v.26.0). Pictures were produced in Excel and PowerPoint. Sample sizes and the replicates were described in the figure legends.

### Reporting summary

Further information on research design is available in the Nature Portfolio Reporting Summary linked to this article.

## Supplementary information

Supplementary Information

Description of Additional Supplementary Files

Supplementary Data 1

Reporting Summary

## Data Availability

Both DNA and RNA sequencing data are available in the Sequence Read Archive (SRA) using accession: PRJNA984921. The source data to generate plots can be found in Supplementary Data 1. The uncropped images of immunoblotting can be found in Supplementary Fig. 4. The gating strategy can be found in Supplementary Fig. 5. Other data are available from the corresponding author on reasonable request.
