# Supplementary material for: Crosstalk between KIF1C and PRKAR1A in left atrial myxoma
Source: Commun Biol. 2023 Jul 14;6:724. doi: 10.1038/s42003-023-05094-5 (PMC10349109; doi:10.1038/s42003-023-05094-5)
Supplement: Supplementary file 1 — Supplementary Information [file 42003_2023_5094_MOESM1_ESM.pdf]

Supplementary information:

Supplementary Figure 1: Sequencing peak figures of rare exon variations of *PRKAR1A* in 28 patients with CM.

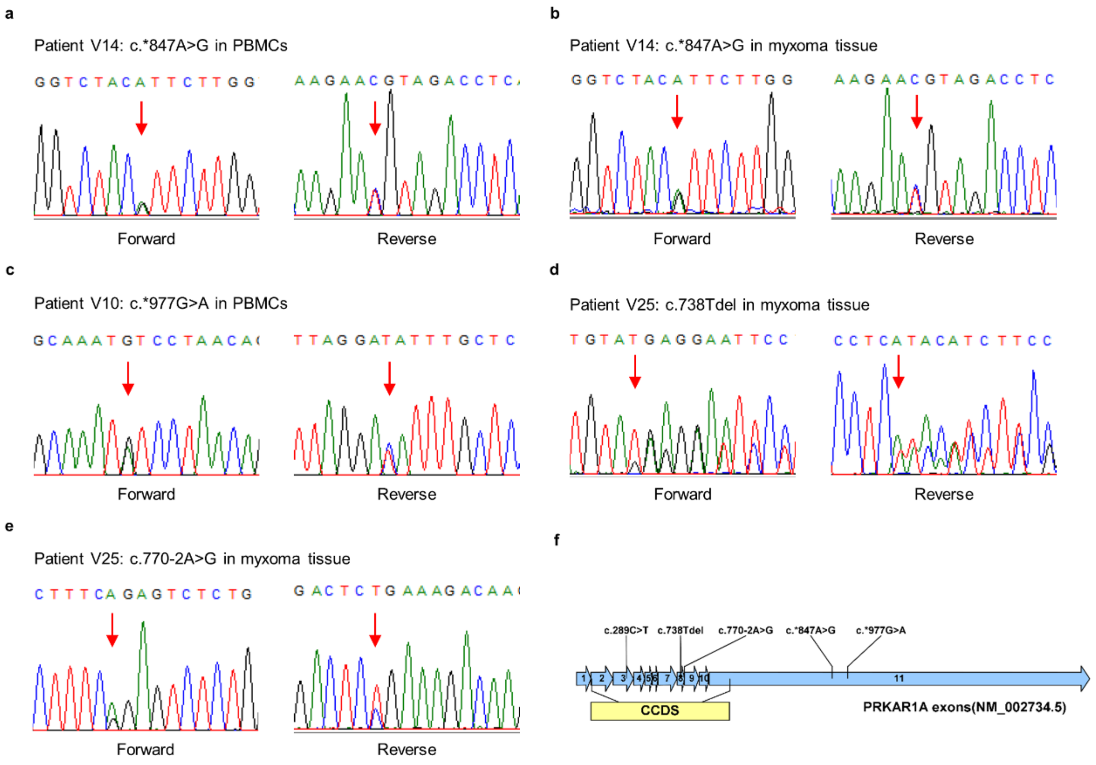

a Sanger sequencing of c.*847A>G in PBMCs of patient V14. **b** Sanger sequencing of c.*847A>G in myxoma tissue of patient V14. **c** Sanger sequencing of c.*977G>A in PBMCs of patient V10. **d** Sanger sequencing of c.738Tdel in myxoma tissue of patient V25. **e** Sanger sequencing of c.770-2A>G in myxoma tissue of patient V25. **f** Distribution of identified *PRKAR1A* variations by Sanger sequencing.

Supplementary Figure 2: Sequencing peak figures of c.*442_*443dup variations in *KIF1C*

a

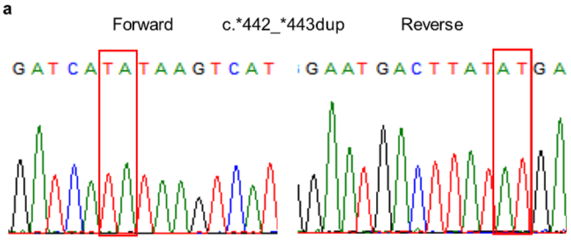

b

Rare variations in 3'UTR of *KIF1C* in left atrial myxoma tissues

No.	Nucleotide change (NM_006612.6)	Protein change (NP_006603.2)	SNV	Frequency						VT
				Korea 1K	TOPMED	ExAC	GnomAD_exome	GnomAD v2.1.1	GnomAD v2.1.1_East Asian	
V7	c.*442_*443dup	3'UTR	rs146142462	NA	0.000737	NA	0.0131	0.0007976	0.01607	Hom

Hom, Homozygous. NA, Not available

a Sanger sequencing of variation c.*442_*443dup in 3'UTR. **b** The minor allele frequency (MAF) of the c.*442_*443dup variation.

Supplementary Figure 3: ***KIF1C*** **variated in various cancer and its mRNA level was decreased.**

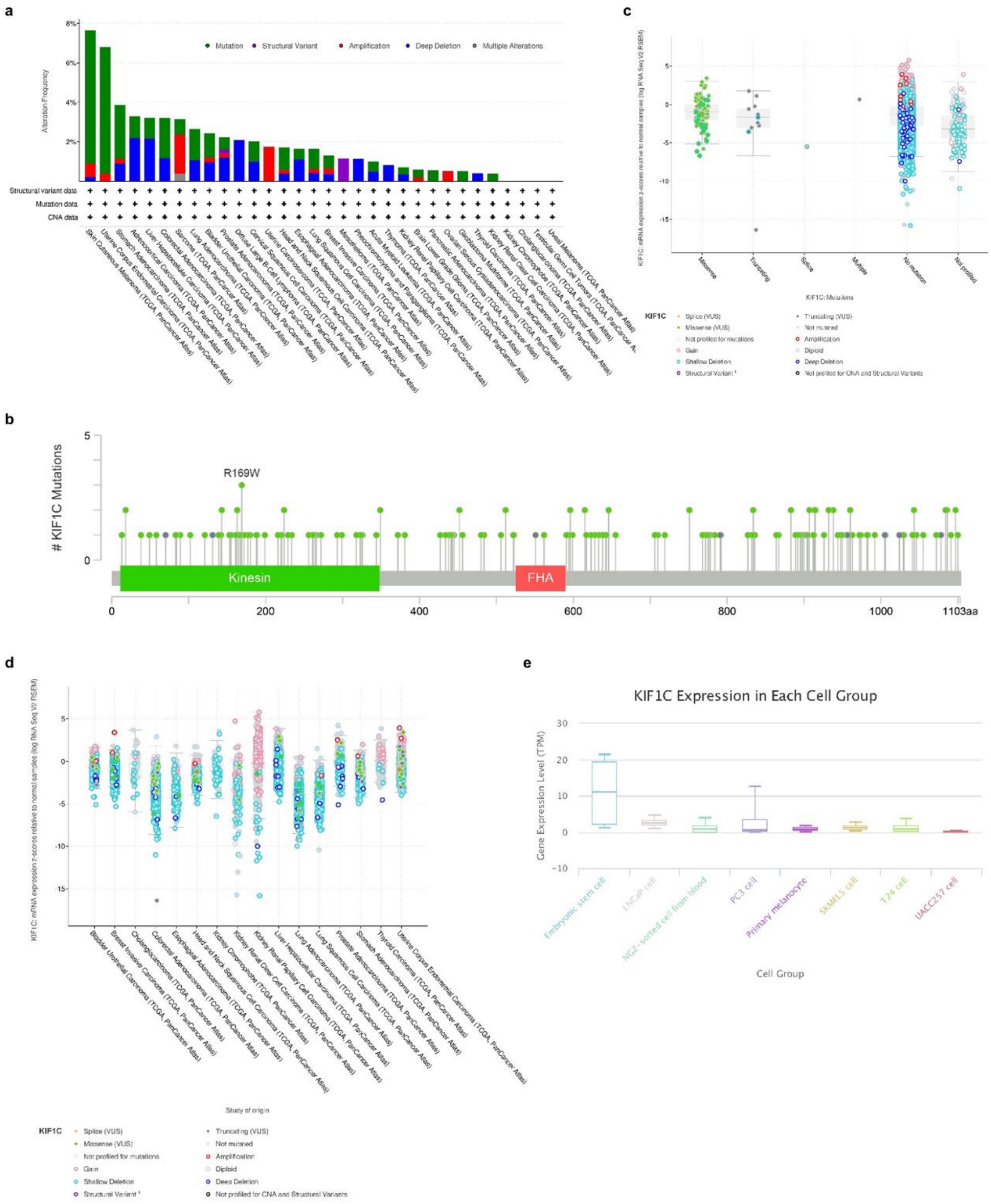

a Mutation, structural variation, amplification, deep deletion, and multiple alterations of *KIF1C* in 10,967 samples from 32 cancer types in the TCGA database. Mutations are depicted in green, amplifications in red, structural variations in purple, deep deletions in blue, and multiple alterations in gray. Plots were obtained from cBioPortal (www.cBioPortal.org). **b** Spectrum of *KIF1C* mutations in 10,967 samples from 32 cancer types in the TCGA database. Diagrams representing the protein domains of KIF1C. The

mutations are shown on the x-axis, and the height of each circle indicates the frequency of the mutation (y-axis). Missense mutations are depicted as green circles, and truncating mutations are depicted as gray circles. Plots were obtained from cBioPortal (www.cBioPortal.org). **c** Diagrams representing the mRNA expression z-scores of *KIF1C* in different *KIF1C* variation types relative to normal samples. Plots were obtained from cBioPortal (www.cBioPortal.org). **d** Diagrams representing the mRNA expression z-scores of *KIF1C* in different cancer types (TCGA) relative to normal samples. Plots were obtained from cBioPortal (www.cBioPortal.org). **e** The mRNA level of *KIF1C* in different cancer cells in a full-length mRNA-Seq dataset (GSE38495). The expression of genes was represented by TPM (transcripts per kilobase of exon model per million mapped reads). Plots were obtained from the single-cell sequencing database scRNASeqDB (https://bioinfo.uth.edu/scrnaseqdb/).

Supplementary Figure 4: Uncropped scans of Western blot results.

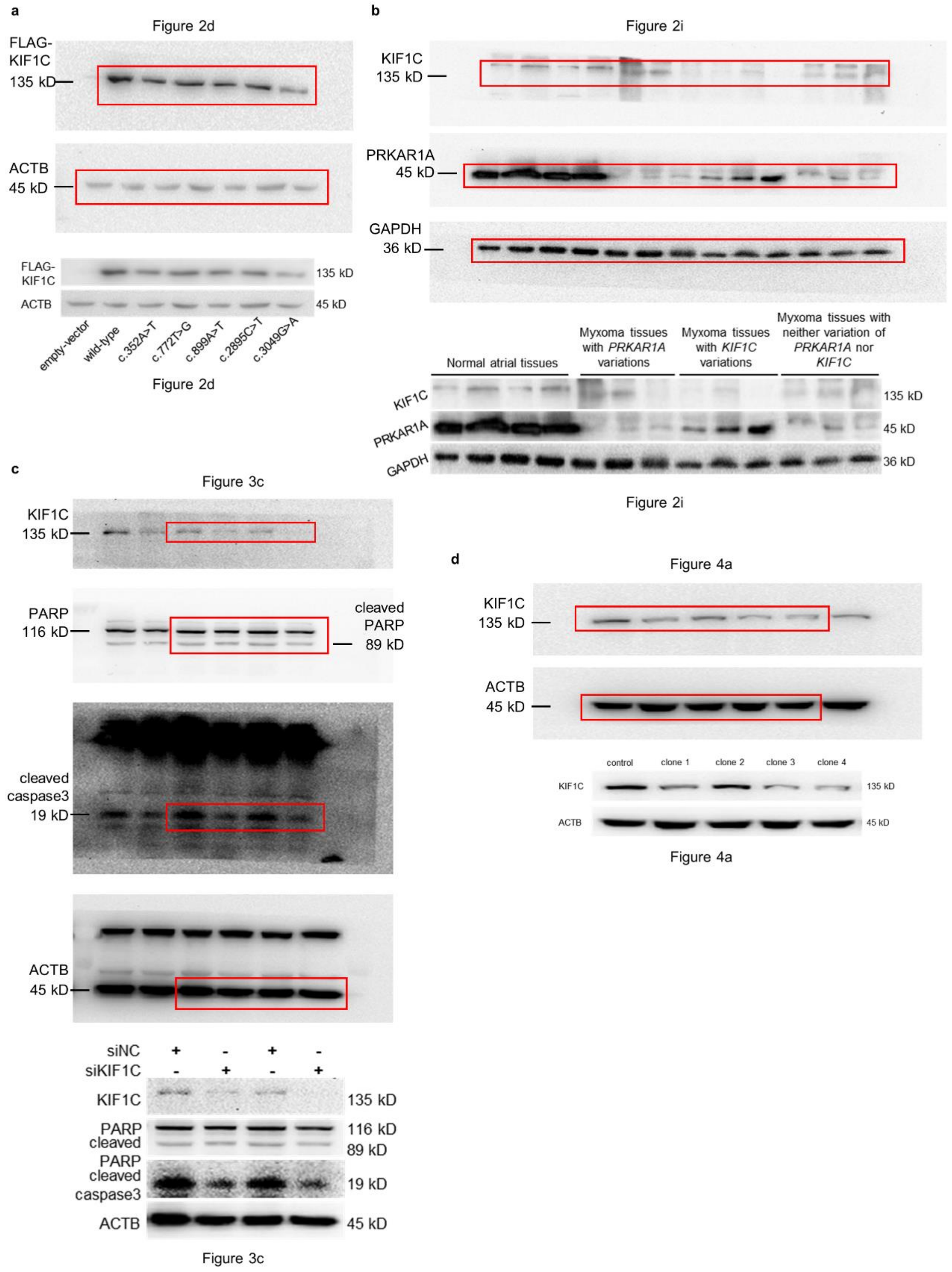

e

Figure 5a

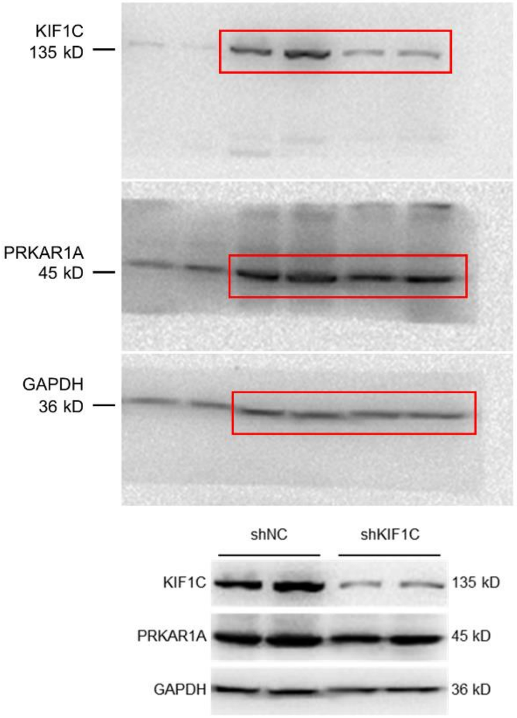

Figure 5a

f

Figure 5c

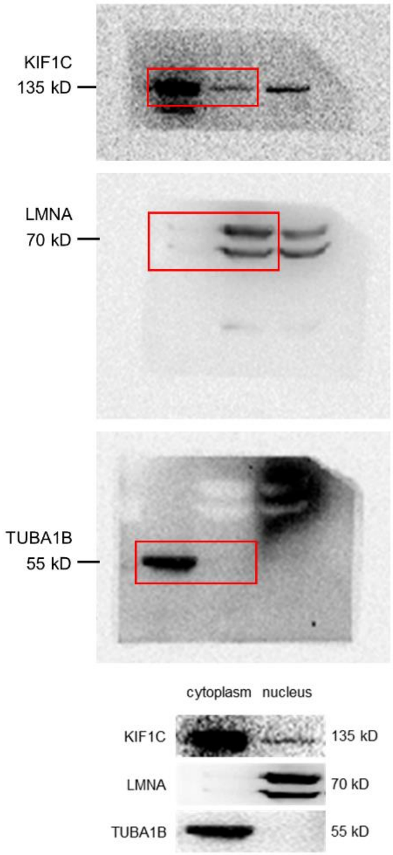

Figure 5c

61

g

Figure 6a

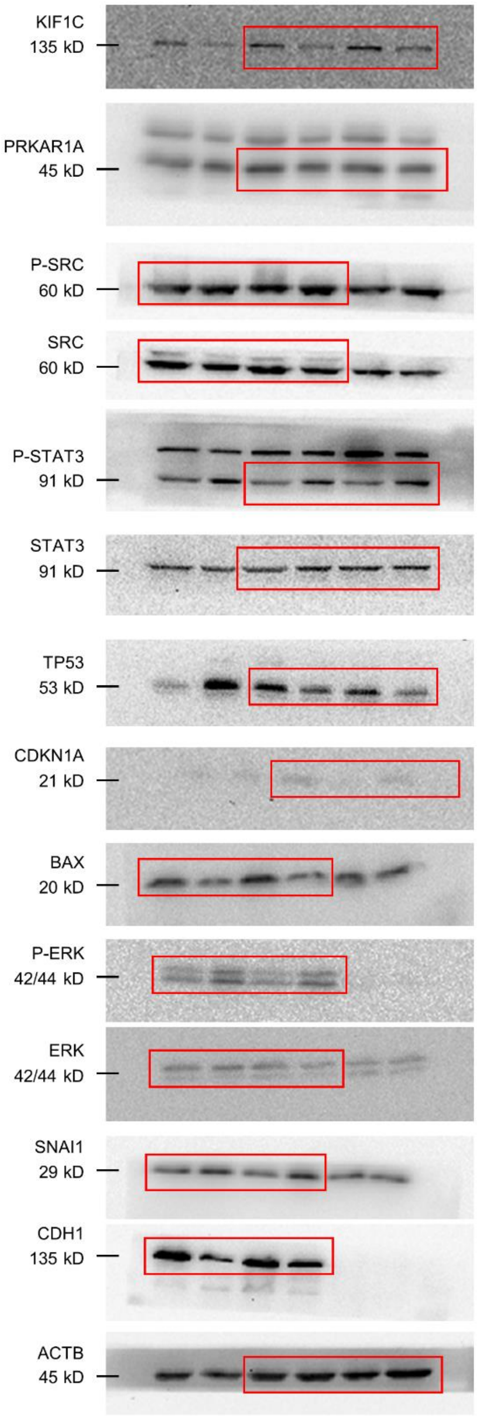

Figure 6a

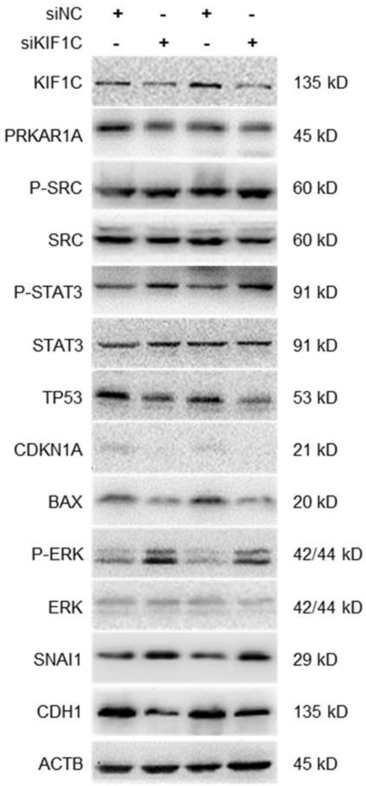

62

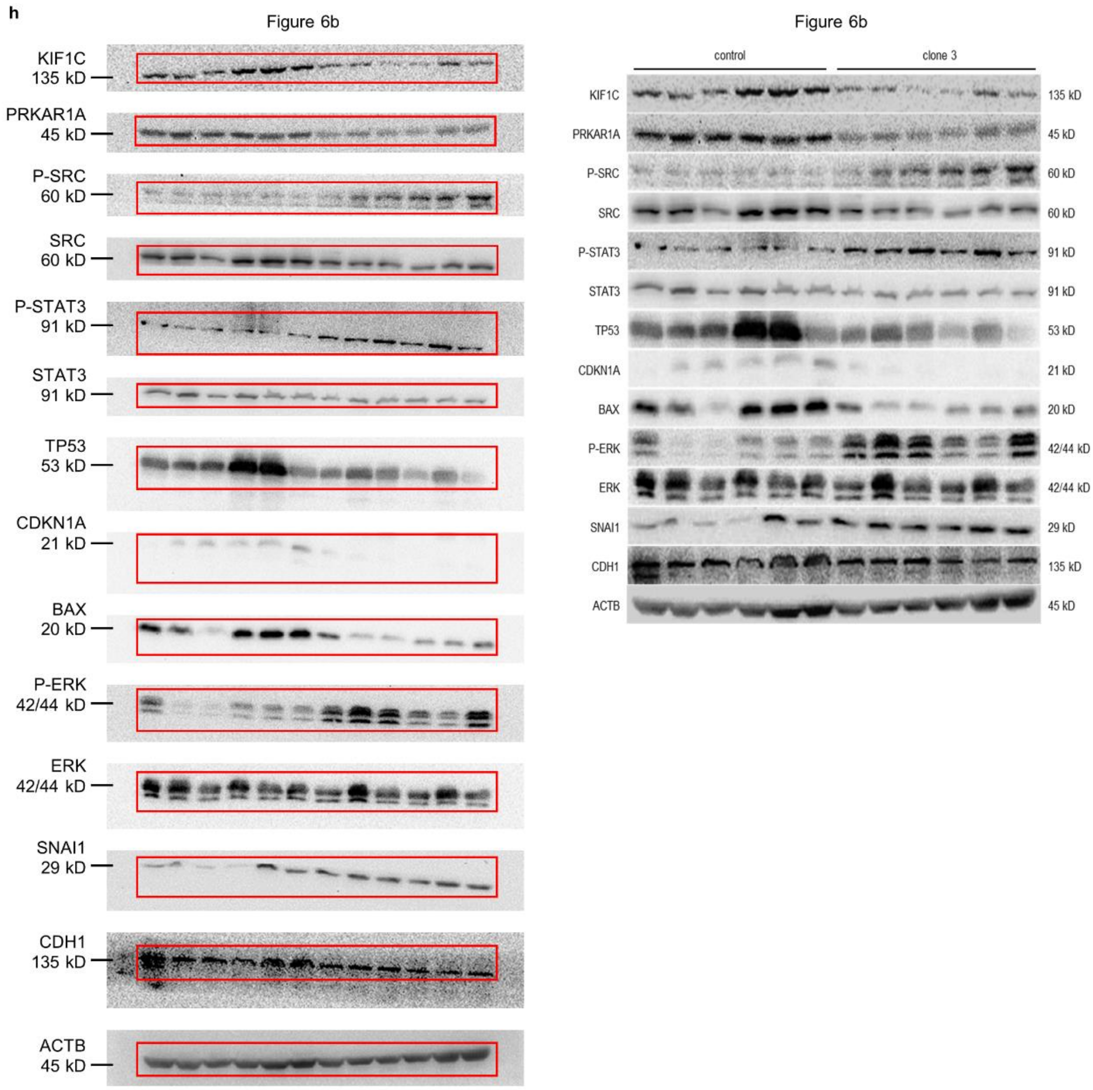

Supplementary Figure 5: The gating strategy in flow cytometry.

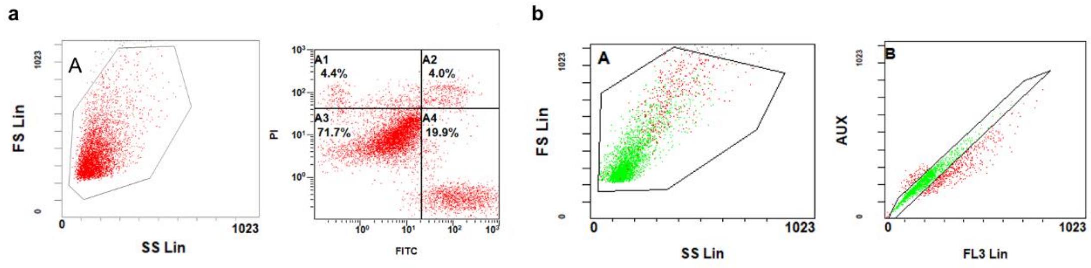

a The cells in gate A were used for apoptosis analysis; the cells in gate A4 were in the early stage of apoptosis, the cells in gate A2 were in the late stage of apoptosis, and they were all calculated as cells in apoptosis. **b** The cells in gate A were used for cell cycle analysis; the cells in gate B were usually single cells.

Supplementary Table 1. Rare exon variations of *PRKAR1A* in 28 patients with CM

type	position	No.	variation	region	Variation Type	function	SNV	Frequency (GnomAD v3.1.1)	Frequency (GnomAD_East Asian v3.1.1)	anotation
B	chr17:68522867	V1[a]	c.289C>T	exon3	Het	stop gained	rs1555813217	0.000006594	0	CM043065/COSV62236002
B	chr17:68531426	V10	c.*977G>A	exon11	Het	3’UTR	rs946354014	0.00003285	0.0005769	-
B	chr17:68531296	V14	c.*847A>G	exon11	Het	3’UTR	rs144299673	0.001242	0.02540	-
T	chr17:68522867	V1[a]	c.289C>T	exon3	Het	stop gained	rs1555813217	0.000006594	0	CM043065/COSV62236002
T	chr17:68531296	V14	c.*847A>G	exon11	Het	3’UTR	rs144299673	0.001242	0.02540	-
T	chr17:68527869	V25	c.738Tdel	exon8	Het	stop gained	-	-	-	-
T	chr17:68528868	V25	c.770-2A>G	splicing	Het	splice acceptor	-	-	-	-

B, Peripheral blood mononuclear cells (PBMCs); T, Myxoma tissue; Het, heterozygous; a, We have reported this before. -: Null.

Supplementary Table 2. The Ct values of three duplicate holes in qRT-PCR of *CYP1A2*

Hole	Normal atrial tissue	Myxoma tissue of patient D1	Myxoma tissue of patient D2
Duplicate 1	Undetermined	37.123	Undetermined
Duplicate 2	30.998	Undetermined	36.559
Duplicate 3	Undetermined	Undetermined	31.208

Supplementary Table 3. Clinical characteristics of 2 patients with LAM for the discovery study

No.	Sample type	Sex	Age (yr)	HR	HTN	CHD	DM	CI	RHD	LAD (mm)	MS (mm)	Mitral valve	Other valves	EF	Ultrasound diagnosis	Pathological diagnosis
D1	T	F	38	SR	0	0	0	0	0	63	81×39	Functional mild stenosis	Severe tricuspid regurgitation	65%	Left atrial space occupying, severe pulmonary hypertension, enlarged atria, pericardial effusion	Left atrial myxoma, myocardial tissue is visible on the pedicle
D2	B & T	M	58	SR	0	0	0	0	0	39	24×20	Mild regurgitation	-	60%	Left atrium mass echo	Left atrial myxoma, myocardial tissue is visible on the pedicle

B, Peripheral blood mononuclear cells (PBMCs); T, Myxoma tissue; F, Female; M, Male; HR, Heart rhythm; SR, Sinus rhythm; HTN, Hypertension; CHD, Coronary heart disease; DM, Diabetes mellitus; CI, Cerebral infarction; RHD, Rheumatic heart disease; LAD, Left atrial diameter; MS, Myxoma size; EF, Ejection fraction; -, Normal; 0, Negative.

Supplementary Table 4. Clinical characteristics of 26 patients with CM for the validation study

No.	Sample type	Sex	Age (yr)	HR	HTN	CHD	DM	CI	RHD	LAD (mm)	MS (mm)	Mitral valve	Other valves	EF	Ultrasound diagnosis	Pathological diagnosis
V1	B & T	M	30	SR	0	0	0	0	0	50	23×20	-	-	54%	Left ventricular space occupying	Left ventricular myxoma, myocardial tissue is visible on the pedicle
V2	B & T	M	66	SR	1	0	0	0	0	38	35×25	Mild regurgitation	-	58%	Left atrial space occupying, thickening of the ventricular septum, Decreased left ventricular diastolic function	Left atrial myxoma, myocardial tissue is visible on the pedicle
V3	T	M	68	SR	1	0	1	1	0	36	22×34	-	-	65%	Left atrial space occupying	Left atrial myxoma, myocardial tissue is visible on the pedicle

V4	B	F	70	AF	0	0	0	0	0	31	33×60	Mild regurgitation	-	64%	Left atrial space occupying	Left atrial myxoma, no tumor infiltration in the basal myocardium
V5	T	F	67	SR	1	1	0	0	0	37	26×20	-	-	70%	Left atrial space occupying, Decreased left ventricular diastolic function	Left atrial myxoma, with bleeding, myocardial tissue is visible on the pedicle
V6	B	M	72	SR	1	0	0	0	0	37	54×52	-	Mild tricuspid regurgitation	62%	Left atrial space occupying	Left atrial myxoma, myocardial tissue is visible on the pedicle
V7	T	F	37	SR	0	0	0	0	0	57	70×34	Severe regurgitation	Mild tricuspid regurgitation	65%	Left atrial space occupying, enlarged left atrium, mild pulmonary hypertension	Left atrial myxoma, with hemorrhagic necrosis
V8	B	F	44	SR	0	0	0	0	0	44	43×56	Mild regurgitation	Mild tricuspid regurgitation	67%	enlarged left atrium, mild pulmonary hypertension	Left atrial myxoma, myocardial tissue is visible on the pedicle

V9	B	M	54	SR	0	0	0	0	0	36	18×25	-	-	66%	Left atrial space occupying, ascending aorta widening, decreased left ventricular diastolic function	Left atrial myxoma, with degeneration necrosis, myocardial tissue is visible on the pedicle
V10	B	M	43	SR	0	0	0	0	0	37	22×21	-	-	68%	Right ventricular apical space occupying	Right ventricular myxoma, myocardial tissue is visible on the pedicle
V11	T	M	65	SR	1	0	1	0	0	46	29×17	Mild regurgitation	-	59%	Left atrial space occupying, enlarged left atrium, thickening of the ventricular septum, decreased left ventricular diastolic function	Left atrial myxoma, myocardial tissue is visible on the pedicle
V12	T	F	70	SR	1	0	1	0	0	37	22×25	Mild regurgitation	-	69%	Left atrial space occupying, decreased left	Left atrial myxoma, myocardial tissue is visible on the pedicle

															ventricular diastolic function	
V13	T	F	69	SR	0	0	0	0	0	40	32×28	Mild regurgitation	Mild tricuspid regurgitation	54%	Left atrial space occupying, left ventricular outflow tract obstruction, thickening of the ventricular septum, enlarged left atrium, decreased left ventricular diastolic function	Left atrial myxoma, myocardial tissue is visible on the pedicle
V14	B & T	F	55	SR	1	0	0	0	0	35	11×13	Anterior mitral leaflet prolapse and severe regurgitation	Mild aortic regurgitation	64%	Left atrial space occupying, enlarged left atrium, ascending aorta widening, decreased left ventricular diastolic function	Left atrial myxoma, myocardial tissue is visible on the pedicle

V15	B	F	54	SR	0	0	0	1	0	34	30×17	-	-	70%	Left atrial space occupying	Left atrial myxoma, myocardial tissue is visible on the pedicle
V16	B & T	F	61	SR	1	0	0	1	0	38	31×20	Mild regurgitation	Mild tricuspid regurgitation	71%	Left atrial space occupying, enlarged left atrium, decreased left ventricular diastolic function	（Left atrial myxoma, myocardial tissue is visible on the pedicle
V17	T	F	63	SR	0	0	0	0	0	44	12×9.5	Mild regurgitation	Mild aortic regurgitation	54%	Left atrial space occupying, ascending aorta widening, enlarged left atrium	Left atrial myxoma, with hemorrhagic necrosis, myocardial tissue is visible on the pedicle
V18	B	F	42	SR	0	0	0	0	0	49	17×13	Mild regurgitation	Mild tricuspid regurgitation	61%	Left atrial space occupying	Left atrial myxoma ， Myocardial tissue is visible on the pedicle

V19	T	M	32	SR	0	0	0	0	0	30	36×21	Mild regurgitation with secondary stenosis	Mild tricuspid regurgitation	64%	Left atrial space occupying	Left atrial myxoma, myocardial tissue is visible on the pedicle
V20	T	F	59	AF	0	0	0	0	0	42	58×29	Secondary mitral valve orifice obstruction	Mild tricuspid regurgitation	68%	Left atrial space occupying, enlarged atria	Left atrial myxoma, myocardial tissue is visible on the pedicle
V21	B	M	64	SR	0	0	0	0	0	55	28×15	Mild regurgitation	Mild tricuspid regurgitation	66%	Left atrial space occupying, enlarged left atrium	Left atrial myxoma, myocardial tissue is visible on the pedicle
V22	B	F	58	SR	0	0	0	0	0	43	62×36	Mild regurgitation， orifice obstruction	Moderate tricuspid regurgitation	68%	Left atrial space occupying, moderate pulmonary hypertension, enlarged left atrium	Left atrial myxoma, myocardial tissue is visible on the pedicle

V23	B	M	70	SR	1	1	0	0	0	50	24×32	Mild regurgitation	Mild tricuspid regurgitation	54%	Left atrial space occupying	Left atrial myxoma, myocardial tissue is visible on the pedicle
V24	B	F	49	SR	0	0	0	1	0	53	62×37	Mild regurgitation	Mild tricuspid regurgitation	63%	Left atrial space occupying, enlarged left atrium	Left atrial myxoma, myocardial tissue is visible on the pedicle
V25	B & T	M	45	SR	0	0	0	0	0	34	35×25	-	-	65%	Left atrial space occupying, decreased left ventricular diastolic function	Left atrial myxoma
V26	B	F	45	SR	0	0	0	0	0	35	40×23	-	-	59%	Left atrial space occupying, decreased left ventricular diastolic function	Left atrial myxoma, myocardial tissue is visible on the pedicle

B, Peripheral blood mononuclear cells (PBMCs); T, Myxoma tissue; F, Female; M, Male; HR, Heart rhythm; SR, Sinus rhythm; AF, Atrial fibrillation; HTN, Hypertension; CHD, Coronary heart disease; DM, Diabetes mellitus; CI, Cerebral infarction; RHD, Rheumatic heart disease; LAD, Left atrial diameter; MS, Myxoma size; EF, Ejection fraction; -, Normal; 0, Negative; 1, Positive.

Supplementary Table 5. Sequences of PCR primers and siRNA

Name	Sequence (5’-3’)
PRKAR1A (ENST00000392711.5) exon amplification and sequencing primers	
Primer 1F	ACCGGATGACTGACCTGAGCC
Primer 1R	CGGTTCTCTCCTCCTTCCTGCTC
Primer 2F	AGAGCGACCAGGGGGGAGGAACT
Primer 2R	CGCTCCCCGTGACGCCATCTT
Primer 3F	TGGGCGTTGGCTTTGGTGC
Primer 3R	TCTGGGAAATAAATAAAAACACTT
Primer 4F	GTAAGAATTCTCCCCTCCCCCAACTAA
Primer 4R	CGCAACAAGTCCTGACCAATAAAATG
Primer 5F	GGAAGAAGAGATTGGAAGTGACTGAG
Primer 5R	GATGAAGTTCCACCCTGTTTTGTATT
Primer 6F	GCTGTAGGCAAGGGGATTAATTAGTCA
Primer 6R	AAAAAAAAAAGTGGTAGGATCAAAGAG
Primer 7F	GTTGCTTGATTTTCTTTCCCCTGA
Primer 7R	CCAGCTACACACACTTCTTCACCAC
Primer 8F	TTTTTTGATGTCACTTGCACTTTAGG
Primer 8R	GACCAGCACATATATACCAGATCTTG
Primer 9F	TTCAGAGATAAATTGGGTTGGCAGTG
Primer 9R	TTTTCCCAAGTCCATCCAATTCTAAT
Primer 10F	TTGGAACGATATGGATGGGGAAACTA

Primer 10R	GGCAGAAAACCAAACAACAGCATT
Primer 11F	CCCTGGGTTTGAGAGTGTGTGTTTGT
Primer 11R	GGCGCTGCAAGTAGGGAAAATAAAG
Primer 12F	TCTGCCTGGTTTTATTTATATCTTGTT
Primer 12R	TACCCTACCATCAACCCACCAGAAAT
Primer 13F	CTGGGGAAGAGGTTTTATTTACATTT
Primer 13R	TCGGGGAAGGACAGAGAGAGGAAAGA
Primer 14F	TTTCTTCCCTTTCTCCTTTCCGTCTT
Primer 14R	AACATACACTGCCAGGGCCTAACA
Primer 15F	GCTCGTGGCCCCTTGAAGTGCGTTAA
Primer 15R	CCGCCTGGCATTTCTCCCTTTAGCA
Primer 15sF (polyT sequencing primer)	ATCCCAGCACCTATTGAA
Primer 16F	TGGGGCATGAGATTTTGGAAGAAGTT
Primer 16R	AGGGAGTAAACAGCAAGCCAAATGAT
Primer 17F	TTGGCTTGCTGTTTACTCCCTTCTG
Primer 17R	TGACAAACTGAAAAGCACTGAAAATAAA
Primer 18F	GCAGCTGATCCACTCCAACCCCTTCT
Primer 18R	GCAAACAGCAGACCCAGGATTCAAAC
Sequencing primers for candidate gene variation sites	
rs200789139-F	CCCAGTCTGTTCCCTTCTCGGCCAC
rs200789139-R	AGGGCCTGCCGGATGGTGTC

rs55918015-F	GTGGGAGGTTAGTGGGGTCGCAGA
rs55918015-R	TGGGGGATGGTGAAGGGCAAGA
rs770752941-F	GCCCTTGCCAGCTGCCCACATTC
rs770752941-R	GCCCCACAACAAACCACCCTCTG
rs760721111-F	CGGCCCTTGCAGATATGGTGAGAC
rs760721111-R	CGGGATTTGGGATTCTCGGCATAG
Sequencing primers for *KIF1C* cDNA（NM_006612.6）	
Primer 1-F	CCGCCGCGCCGAGGGTATC
Primer 1-R	CGAAGTGTGTGACCAGTAGGAGTAGT
Primer 2-F	GGGCAACACCACCTCCATCATCAA
Primer 2-R	ATGGGAACGGCTGCTGGTCTCA
Primer 3-F	GTCGGGGTTCTCTGCGGGTCC
Primer 3-R	GGCTCCAGCTCCCCATTATGTGTG
Primer 4-F	CCCCAGCTCCAGTTTCACCCTCAT
Primer 4-R	CAAGGCTCCAGAGTGACCACCA
Primer 5-F	GCGTCACCAGGGTCGGCCAAGTAGA
Primer 5-R	GGCAGACCACAGCGTTTGACAA
Primer 6-F	CGGGGATGACTCTGACAAGCGCTC
Primer 6-R	TCCCGGTCCTTGCTGCTGTTCTG
Primer 7-F	GCCCTGGCCGCCCTCAAGATG
Primer 7-R	TGGCCAAGACTCCCGGTGCTG
Primer 8-F	CCCCCCTCGCCACCACTGTCAA

Primer 8-R	GCTGCGCACCCTGAAGGAAGAGAC
qRT-PCR primers	
PRKAR1A-RT-F	GGGGCATCGACCGAGACAGCTATA
PRKAR1A-RT-R	GGCCCCAATCTTCCCACTTCAAC
KIF1C-RT-F	CCGCCGCGCCGAGGGTATCT
KIF1C-RT-R	GGGGGTCCTCCGTCGAAGTGTGT
ACTB-RT-F	ATCCGCCGCCCGTCCACA
ACTB-RT-R	CATGGCTGGGGTGTTGAAGGTCT
BAX-RT-F	TGCCGCCGTGGACACAGACT
BAX-RT-R	CAGGGCCTTGAGCACCAGTTTG
TP53-RT-F	ACCTATGGAAACTACTTCCTGAAA
TP53-RT-R	CTGGCATTCTGGGAGCTTCA
CDKN1A-RT-F	GGCTCCTTCCCATCGCTGTCACA
CDKN1A-RT-R	CCACCTTCCCCCTGCCTTCACAA
CDH1-RT-F	GCCGCCATCGCTTACACCATCCTCAG
CDH1-RT-R	TGGGGTCTTGGGGGCATCAGCATCA
SRC-RT-F	GCCCCACTCTGCCTGCCTGCTGTT
SRC-RT-R	GGGGCCCATCGCTCCTCTTTG
ChIP primers	
ChIP 1-F	GGCTTCTGCATTCTTCACGTAG
ChIP 1-R	CGAGCTGAGAGAAGAAGGCTTC
ChIP 2-F	TGCCCGTTCTCAGATCTCCAGCT

ChIP 2-R	CCGGGAAGCTCGAACTGGGTGGA
ChIP 3-F	ATCTGTCTAATGTTGACAGTGG
ChIP 3-R	GATCTTTGTTGGTTTAAAGTCTG
Primers for construction of plasmids	
pGL6-*PRKAR1A* promotor-F	GGGGTACCTCAAAGTTATTCTCTGTCCAGCT
pGL6-*PRKAR1A* promotor-R	CCCAAGCTTCGGCGCACCAGGAGATTACAT
pLVX-shKIF1C-puro F	GATCCCACCGCCGTCTTTACCATCGTCTTTCAAGAG<br>AAGACGATGGTAAAGACGGTTTTTTGGATCG
pLVX-shKIF1C-puro R	AATTCGATCCAAAAAACCGTCTTTACCATCGTCTTC<br>TCTTGAAAGACGATGGTAAAGACGGCGGTGG
pLVX-shNC-puro F	GATCCCACCGTTCTCCGAACGTGTCACGTCAAGAG<br>ATTACGTGACACGTTCGGAGAATTTTTTGGATCG
pLVX-shNC-puro R	AATTCGATCCAAAAAATTCTCCGAACGTGTCACGT<br>AATCTCTTGACGTGACACGTTCGGAGAACGGTGG
pCMV-3×Flag-KIF1C-WT-F	CCGGAATTCAATGGCTGGTGCCTGCCTGAAAGT
pCMV-3×Flag-KIF1C-WT-R	GGGGTACCTCACACAGCTGCCCCACTCTCCTT
pCMV-3×Flag-KIF1C-Mut1-F	ACAGGAGCCAGGGCAGCAGGGCTTCGTGCCCCA
pCMV-3×Flag-KIF1C-Mut1-R	TCCTCACAGAGCTGGGGCACGAAGCCCTGCTGC
pCMV-3×Flag-KIF1C-Mut2-F	TGGGAGTGAGCGAGCCGACTCCGCAGGGGCCCG
pCMV-3×Flag-KIF1C-Mut2-R	AGGCGCATGCCCCGGGCCCCTGCGGAGTCGGCT
pCMV-3×Flag-KIF1C-Mut3-F	GCGAAAGTCGGATTTTATCCCCTTCAGGGACTCT
pCMV-3×Flag-KIF1C-Mut3-R	GCCAGGTGAGCACAGAGTCCCTGAAGGGGATAAA

pCMV-3×Flag-KIF1C-Mut4-F	CCGGGGCGGGGGGCTGCGCAGGCCTCCAGCCCGCT
pCMV-3×Flag-KIF1C-Mut4-R	TGAGGGGGCACAAAGCGGGCTGGAGGCCTGCGCA
pCMV-3×Flag-KIF1C-Mut5-F	TCACTCCCCATCCAGCCACCCCTACCCGCCGGCCT
pCMV-3×Flag-KIF1C-Mut5-R	TTCGGGGACTCGGAGGCCGGCGGGTAGGGGTGGCT
siRNA sequences	
Negative Control siRNA	UUC UCC GAA CGU GUC ACG UTT
KIF1C Homo siRNA	CCG UCU UUA CCA UCG UCU UTT

Mut1: c.352A>T; Mut2: c.772T>G; Mut3: c.899A>T; Mut4: c.2895C>T; Mut5: c.3049G>A

Supplementary Table 6. Key Resources Table

REAGENT or RESOURCE	SOURCE	IDENTIFIER
Antibodies		
KIF1C	Novus Biologicals	NBP1-85978
PRKAR1A	Proteintech	20358-1-AP
E-cadherin (G-10)	Santa Cruz Biotechnology	Sc-8426
Snail	ABclonal Technology	A11794
p53 (DO-1)	Santa Cruz Biotechnology	sc-126
p21 (F-5)	Santa Cruz Biotechnology	sc-6246
Bax (D2E11)	Cell Signaling Technology	5023S
β-Actin（13E5）	Cell Signaling Technology	#5057s
Gapdh	ABMART	M20006
α-tubulin	Proteintech	11224-1-AP
Lamin A/C	Proteintech	10298-1-AP
src	ABclonal Technology	A19119
Phospho-Src Family (Tyr416) (D49G4)	Cell Signaling Technology	#6943
Phospho-p44/42 MAPK（Erk1/2） (Thr202/Tyr204)	Cell Signaling Technology	#9101s
p44/42 MAPK (Erk1/2)	Cell Signaling Technology	#9102s
Cleaved caspase 3（Asp175）(5A1E)	Cell Signaling Technology	#9664s

PARP	Cell Signaling Technology	#9542s
Cleaved PARP（Asp214）(D64E10) XP®	Cell Signaling Technology	#5625s
p-Stat3 (B-7)	Santa Cruz Biotechnology	sc-8059
Stat3 (F-2)	Santa Cruz Biotechnology	sc-8019
Critical Commercial Assays		
SimpleChIP™ Enzymatic Chromatin IP Kit (Magnetic Beads)	Cell Signaling Technology	#9003
Dual-Glo® Luciferase Assay System	Promega	E2920
BD Pharmingen™ FITC Annexin V	BD biosciences	556420
Cell-Light™ EdU Apollo567 In Vitro Kit	Ribobio	C10310-1
Nuclear and cytoplasmic extraction Kit	BestBio	BB-3102-100T
