# Supplementary material for: Crosstalk between KIF1C and PRKAR1A in left atrial myxoma
Source: Commun Biol. 2023 Jul 14;6:724. doi: 10.1038/s42003-023-05094-5 (PMC10349109; doi:10.1038/s42003-023-05094-5)
Supplement: Supplementary file 2 — Description of Additional Supplementary Files [file 42003_2023_5094_MOESM2_ESM.pdf]

Description of Additional Supplementary Files

File name: Supplementary data 1

Description: The source data to generate plots.
