# Supplementary material for: Crosstalk between KIF1C and PRKAR1A in left atrial myxoma
Source: Commun Biol. 2023 Jul 14;6:724. doi: 10.1038/s42003-023-05094-5 (PMC10349109; doi:10.1038/s42003-023-05094-5)
Supplement: Supplementary file 4 — Reporting Summary [file 42003_2023_5094_MOESM4_ESM.pdf]

nature portfolio

Corresponding author(s): Xin Tu

Last updated by author(s): 2023-5-17

Reporting Summary

Nature Portfolio wishes to improve the reproducibility of the work that we publish. This form provides structure for consistency and transparency in reporting. For further information on Nature Portfolio policies, see our Editorial Policies and the Editorial Policy Checklist.

Statistics

For all statistical analyses, confirm that the following items are present in the figure legend, table legend, main text, or Methods section.

n/a	Confirmed	
☐	☒	The exact sample size (*n*) for each experimental group/condition, given as a discrete number and unit of measurement
☐	☒	A statement on whether measurements were taken from distinct samples or whether the same sample was measured repeatedly
☐	☒	The statistical test(s) used AND whether they are one- or two-sided<br>*Only common tests should be described solely by name; describe more complex techniques in the Methods section.*
☐	☒	A description of all covariates tested
☐	☒	A description of any assumptions or corrections, such as tests of normality and adjustment for multiple comparisons
☐	☒	A full description of the statistical parameters including central tendency (e.g. means) or other basic estimates (e.g. regression coefficient) AND variation (e.g. standard deviation) or associated estimates of uncertainty (e.g. confidence intervals)
☐	☒	For null hypothesis testing, the test statistic (e.g. *F*, *t*, *r*) with confidence intervals, effect sizes, degrees of freedom and *P* value noted<br>*Give P values as exact values whenever suitable.*
☒	☐	For Bayesian analysis, information on the choice of priors and Markov chain Monte Carlo settings
☒	☐	For hierarchical and complex designs, identification of the appropriate level for tests and full reporting of outcomes
☒	☐	Estimates of effect sizes (e.g. Cohen's *d*, Pearson's *r*), indicating how they were calculated

Our web collection on statistics for biologists contains articles on many of the points above.

Software and code

Policy information about availability of computer code

Data collection	Excel, CXP software, FV10-ASW 3.1 Viewer, Quantity One, and StepOne software were used for data collection.
Data analysis	Image J/Fiji were used for monoclone and IHC analyses. CXP software and ModFit software were used for flow cytometric data analyses. Excel and SPSS (v.26.0) were used for statistical analyses.

For manuscripts utilizing custom algorithms or software that are central to the research but not yet described in published literature, software must be made available to editors and reviewers. We strongly encourage code deposition in a community repository (e.g. GitHub). See the Nature Portfolio guidelines for submitting code & software for further information.

Data

Policy information about availability of data

All manuscripts must include a data availability statement. This statement should provide the following information, where applicable:

- Accession codes, unique identifiers, or web links for publicly available datasets
- A description of any restrictions on data availability
- For clinical datasets or third party data, please ensure that the statement adheres to our policy

Both DNA and RNA sequencing data are available in the Sequence Read Archive (SRA) using accession: PRJNA984921. The source data to generate plots can be found in Supplementary Data 1. The uncropped images of immunoblotting can be found in Supplementary Figure 4. The gating strategy can be found in Supplementary Figure 5. Other data are available from the corresponding author on reasonable request.

Human research participants

Policy information about studies involving human research participants and Sex and Gender in Research.

Reporting on sex and gender	The human research participants include both males and females.
Population characteristics	All the participants included in the study are Chinese.The detailed age, gender, diagnosis, and other information for each patient were shown in supplementary tables 2 and 3.
Recruitment	Participants were recruited when they visited their doctors.
Ethics oversight	Ethical Review Board of Beijing Anzhen Hospital

Note that full information on the approval of the study protocol must also be provided in the manuscript.

Field-specific reporting

Please select the one below that is the best fit for your research. If you are not sure, read the appropriate sections before making your selection.

☒ Life sciences ☐ Behavioural & social sciences ☐ Ecological, evolutionary & environmental sciences

For a reference copy of the document with all sections, see nature.com/documents/nr-reporting-summary-flat.pdf

Life sciences study design

All studies must disclose on these points even when the disclosure is negative.

Sample size	Two tissue samples, a PBMCs sample, and three control samples were used for WES. Two tissue samples and a control sample were used for RNA-seq. 18 PBMC samples and 16 myxoma tissue samples were used for PRKAR1A mutation screening, and 13 tissue samples were used for KIF1C mutation screening. Six mice were used in each group.
Data exclusions	In the tumor growth assays in vivo, one outlier in each group was excluded.
Replication	There were at least three tissue samples when measuring the expression of KIF1C in each group of patients or controls. There were four replicate wells for each cell line when staining crystal violet. There were six mice as replications in each group in tumor growth assays. Other experiments all had three dependent replications, and they were all successful.
Randomization	BALB/c-nude mice were randomly divided into two groups.
Blinding	The inoculated cells were resuspended by one person, and the data collection on mice was carried out by another person. Measuring the expression of KIF1C to distinguish the groups.

Reporting for specific materials, systems and methods

We require information from authors about some types of materials, experimental systems and methods used in many studies. Here, indicate whether each material, system or method listed is relevant to your study. If you are not sure if a list item applies to your research, read the appropriate section before selecting a response.

Materials & experimental systems

n/a	Involved in the study	
☐	☒	Antibodies
☐	☒	Eukaryotic cell lines
☒	☐	Palaeontology and archaeology
☐	☒	Animals and other organisms
☒	☐	Clinical data
☒	☐	Dual use research of concern

Methods

n/a	Involved in the study	
☒	☐	ChIP-seq
☐	☒	Flow cytometry
☒	☐	MRI-based neuroimaging

Antibodies

Antibodies used	All the detailed information about the antibodies used in the study was provided in Supplementary Table 6.

Validation	The application of KIF1C primary antibody for immunofluorescence and chromatin co-immunoprecipitation was validated in the manuscript. Species and applications for other primary antibodies were validated by the manufactures.

Eukaryotic cell lines

Policy information about cell lines and Sex and Gender in Research

Cell line source(s)	The HCT116 , B16, FMC84, and AC16 cell lines were preserved in our laboratory.
Authentication	HCT116, B16, and AC16 cell lines were authenticated by STR. The species identification of FMC84 was performed by PCR.
Mycoplasma contamination	All the cells tested negative for mycoplasma contamination.
Commonly misidentified lines (See ICLAC register)	The HCT116 cell line preserved in our laboratory was previously used for tumor studies.

Animals and other research organisms

Policy information about studies involving animals; ARRIVE guidelines recommended for reporting animal research, and Sex and Gender in Research

Laboratory animals	BALB/c-nude mice
Wild animals	The study did not involve wild animals.
Reporting on sex	All the mice were female. Sex is determined primarily by the mouse's reproductive system.
Field-collected samples	The study did not involve samples collected from the field.
Ethics oversight	The mouse studies were approved by the Ethical Review Board of Beijing Anzhen Hospital.

Note that full information on the approval of the study protocol must also be provided in the manuscript.

Flow Cytometry

Plots

Confirm that:

☒ The axis labels state the marker and fluorochrome used (e.g. CD4-FITC).

☒ The axis scales are clearly visible. Include numbers along axes only for bottom left plot of group (a 'group' is an analysis of identical markers).

☒ All plots are contour plots with outliers or pseudocolor plots.

☒ A numerical value for number of cells or percentage (with statistics) is provided.

Methodology

Sample preparation	The cells used for detecting apoptosis were stimulated with 150 µM H2O2 for 6 hours and proceeded according to the instructions of the FITC Annexin V Apoptosis Detection Kit. The cells used to detect the cell cycle were stained with propidium iodide.
Instrument	Beckman FC500 flow cytometer
Software	The results were analyzed with CXP software when analyzing apoptosis and ModFit software when analyzing cell cycle.
Cell population abundance	10000 cells were collected for each analysis.
Gating strategy	In the apoptosis analysis, set a gate in FSC/SSC scatters. FITC positive control cells stained with FITC were used to draw the gate, PI positive control cells stained with PI were used to draw the gate, and double positive control cells stained with both FITC and PI were used to draw the gate. In cell cycle analysis, set a gate in FSC/SSC scatter. Draw a scatter plot of FL3 Lin versus AUX, gated on the diagonal, where the cells are usually single cells, to exclude adhesions and cellular debris.

☒ Tick this box to confirm that a figure exemplifying the gating strategy is provided in the Supplementary Information.
